# 17β-estradiol upregulates IL6 expression through the ERβ pathway to promote lung adenocarcinoma progression

**DOI:** 10.1186/s13046-018-0804-5

**Published:** 2018-07-03

**Authors:** Quanfu Huang, Zheng Zhang, Yongde Liao, Changyu Liu, Sheng Fan, Xiao Wei, Bo Ai, Jing Xiong

**Affiliations:** 1 Department of Thoracic Surgery, Tongji Hospital, Tongji Medical College, Huazhong University of Science and Technology, Wuhan, China; 2 Cancer Biology Research Center (Key Laboratory of the Ministry of Education), Tongji Hospital, Tongji Medical College, Huazhong University of Science and Technology, Wuhan, China; 3 Department of Pathology, Tongji Hospital, Tongji Medical College, Huazhong University of Science and Technology, Wuhan, China

**Keywords:** 17β-estradiol, Estrogen receptor β, Interleukin 6, Non-small cell lung cancer

## Abstract

**Background:**

In non-small cell lung cancer (NSCLC), estrogen (E2) significantly promotes NSCLC cell growth via estrogen receptor beta (ERβ). Discovery and elucidation of the mechanism underlying estrogen-promoted NSCLC progression is critical for effective preventive interventions. IL6 has been demonstrated to be involved in the development, progression and metastasis in several cancers and IL6 overexpression is associated with poor prognosis in NSCLC. However, the exact role played by IL6 in estrogen-promoted NSCLC progress remain unknown. Here, we evaluated the expression and biological effects of IL6 in NSCLC cells when treated with E2 and explored the underlying mechanism of IL6 in E2-promoted NSCLC progression.

**Methods:**

Expression of ERβ/IL6 in 289 lung cancer samples was assessed by immunohistochemistry. Matched samples of metastatic lymph node and primary tumor tissues were used to quantify the expression of ERβ/IL6 by western blot. Expression levels of IL6 in NSCLC cells were quantified by western blotting, ELISA, and immunofluorescence staining. The effects of IL6 stimulated by E2 on cell malignancy were evaluated using CCK8, colony formation, wound healing and transwell. Furthermore, overexpression and knockdown ERβ constructs were constructed to measure the expression of IL6. The effects of IL6 stimulated by E2 on tumor growth were evaluated using a urethane-induced adenocarcinoma model. In addition, a xenograft mouse model was used to observe differences in ERβ subtype tumor growth with respect to IL6 expression.

**Results:**

IL6/ERβ expression were significantly increased in lung cancer. Higher IL6/ERβ expression was associated with decreased differentiation or increased metastasis. IL6 was an independent prognostic factor for overall survival (OS), higher IL6 expression was associated with decreased OS. Furthermore, ERβ regulates IL6 expression via MAPK/ERK and PI3K/AKT pathways when stimulated by E2 and promotes cell malignancy in vitro and induced tumor growth in vivo. Finally we confirm that ERβ isolation 1/5 is essential for E2 promotion of IL6 expression, while ERβ2 not.

**Conclusions:**

Our findings demonstrate that E2 stimulates IL6 expression to promote lung adenocarcinoma progression through the ERβ pathway. We also clarify the difference in each ERβ subtype for E2 promoting IL6 expression, suggesting that ERβ/IL6 might be potential targets for prognostic assessment and therapeutic intervention in lung cancer.

**Electronic supplementary material:**

The online version of this article (10.1186/s13046-018-0804-5) contains supplementary material, which is available to authorized users.

## Background

Lung cancer is the most commonly diagnosed cancer as well as the leading cause of cancer death globally in both men and women [1]. Researchers have sought to find new therapeutic targets to improve the efficacy of traditional therapy. Recently, a body of evidence demonstrated that estrogen is a driver of NSCLC. As breast cancers are mostly fueled by estrogen, anti-estrogen treatment is commonly used in breast cancer. A study including 6500 breast cancer survivors found that women who received anti-estrogen treatment exhibited lower lung cancer mortality rates [2]. In the Women’s Health Initiative, more than 16,000 postmenopausal women received a placebo or daily hormone replacement therapy (HRT) for 5 years, and higher lung cancer incidence and mortality rate were found in the HRT group [3]. Additionally, a prospective study demonstrated that HRT might have a duration-dependent effect for increased risk of lung cancer [4]. Therefore, sex-dependent hormones, such as estrogen, may play an important role in the etiology and progression of lung cancer.

Estrogens govern many physiological functions, such as cell growth, development, and differentiation, through estrogen receptor (ER)-mediated signaling in a wide range of tissues [5]. As a specific agonist of ER, E2 induces ER expression and activates the ER pathway. Conventional wisdom suggests that ER comprises two subtypes, namely, ERα and ERβ, which are encoded by separate genes [6]. Although ERα and ERβ have similar structures and ligand-binding patterns, their tissue distribution and relative expression levels are varied [7]. In contrast to breast cancer, ERβ stimulated by estrogen is the major ER subtype found in NSCLC [8–10]. ERβ was shown to be a mediator of estrogen signaling in lung cancer cells, functioning through both genomic and non-genomic mechanisms [11, 12]. Together, these findings suggest that ERβ is the predominant ER in lung tissue and plays an important role in the physiological and pathophysiological functions of the lungs. ERβ is expressed as five protein isoforms, but only ERβ1, ERβ2 and ERβ5 expression has been documented in NSCLC, and the function of each subtype is distinct [13].

Interleukin 6 (IL6) is a pleiotropic cytokine that plays an important role in multiple pathophysiological properties associated with cancer development, including lung cancer [14–17]. The concentration of IL6 in tumor tissue correlates with tumor progression and overall survival in patients with NSCLC [18], and higher postoperative serum IL-6 levels predict a higher risk of early postoperative recurrence [19]. All together, these findings indicate that IL6 does indeed play a role in NSCLC. In endometrial cancer, E2 has been shown to induce IL6 production through cooperation between ERα and nuclear factor-kappa B [20]. Interestingly, activation of the signal transducer and activator of transcription 3 (STAT3) pathway also induces ERβ expression in lung adenocarcinoma cells [21]. However, whether E2 mediates IL6 expression in lung cancer is unknown. We previously reported that E2 promotes lung cancer progression [22]. Other evidence illustrated that IL6 is up-regulated by E2 through the estrogen receptor pathway in endometrial cancer [21, 23], suggesting that IL6 is an important estrogen target gene. Therefore, we hypothesize that crosstalk between the E2 pathway and IL6 may play an important role in the progression of lung cancer.

Therefore, to investigate the relationship between E2, ERβ and IL6 in lung cancer and test the hypothesis that E2 up-regulates IL6 through ERβ to promote lung cancer progression, in the present study, we demonstrate that both IL6 and ERβ expression regulate clinicopathological factors in NSCLC. Immunohistochemistry and western blot by matched metastatic lymph node and primary tumor was performed. Additionally, we evaluated the expression of IL6 under conditions of E2 treatment, ERβ knockdown, ERβ overexpression, and ERβ pathway key molecular inhibition. Furthermore, we utilized two mouse model of NSCLC to detect whether E2 induces IL6 expression by the ERβ pathway and the difference in ERβ isoforms.

## Methods

### Patients and samples

A total of 289 formalin-fixed, paraffin-embedded tissue samples were included in this study, these samples were obtained from patients diagnosed with primary non-small cell lung cancer who underwent initial surgery at the Department of Thoracic Surgery, Affiliated Tongji Hospital of Huazhong University of Science and Technology Tongji Medical College (Wuhan, China) from 2008 to 2015. All the tissue samples were obtained with informed consent from patients. None of the patients had any prior history of chemotherapy, radiation, or hormonal therapy before the surgery. Clinical information of the patients was recorder in detail, and the diagnoses were confirmed by at least two pathologists. pTNM stage and tumor differentiation grade were obtained from the Tongji hospital records. Baseline characteristics of the patients are shown in Table 1. In these patients, metastatic lymph nodes were obtained from 30 cases involving paired primary tumors via surgical resection of primary tumor with lymph node dissection. Cases of lymphadenitis and primary malignancies of lymph node were excluded. Details of the 30 IIA-IIIB NSCLC cases with lymph node metastasis are included in this study (Table 3). Each metastatic lymph node represented the primary tumor tissue referred to above. Palliative care or surgical biopsy following informed consent was administered to 38 cases of patients with inoperable Stage IIIB–IV primary NSCLC.

**Table 1:** The relationship between ERβ/IL6 expression levels and clinical characteristics of NSCLC patients

	No. of patients(%)	ERβ Expression		*χ2*	*p* value	IL6 Expression		*χ2*	*p* value
		Positive	Negative			Positive	Negative		
All	289	258	31			270	19		
Gender									
Female	102(35.29)	95	7	2.458	0.117	99	3	3.388	0.066
Male	187(64.71)	163	24			171	16		
Age									
< 60	170(58.82)	150	20	0.465	0.495	158	12	0.158	0.691
≥ 60	119(41.18)	108	11			112	7		
Smoking									
Ex	178(61.59)	162	16	1.462	0.227	171	7	5.266	0.022
Never	111(38.41)	96	15			99	12		
T stage									
1a-2b	220 (76.12)	194	26	1.146	0.284	205	15	0.089	0.765
3–4	69 (23.88)	64	5			65	4		
Lymph node metastasis									
YES	187(64.71)	167	20	0.001	0.974	178	9	2.677	0.102
NO	102(35.29)	91	11			92	10		
Metastasis									
YES	24(08.30)	18	6	5.568	0.018	20	4	4.34	0.037
NO	265(91.70)	240	25			250	15		
TNM stage									
I-II	175 (60.55)	154	21	0.751	0.386	162	13	0.527	0.468
III-IV	114(39.45)	104	10			108	6		
Tumor Histology									
SQC	134(46.37)	115	19	3.11	0.078	125	9	0.008	0.92873
ADC	155(53.63)	143	12			145	10		
Tumor Differentation									
Well/Moderate	156(53.98)	134	22	4.034	0.045	141	15	5.104	0.024
Poor	133(46.02)	124	9			129	4		

*P* values listed are derived from χ2 test

This study was approved by the Research Ethics Committee, Tongji Medical College, Huazhong University of Science and Technology (The IRB ID number is 20141101).

### Tissue microarray (TMA) preparation

Samples were formalin-fixed, paraffin-embedded, and then diagnosed and confirmed by at least two lung cancer pathologists. TMA was prepared by Outdo Biotech Co. Ltd. (Shanghai, China).

### Antibodies

Human anti-p-ERK, anti-ERK, anti-AKT, anti-p-AKT and anti-p-Stat3 were purchased from Cell Signaling Technology (Danvers, MA, USA). Human anti- ERβ, anti-Bcl-XL, anti-GAPDH (Proteintech, CA, USA). Human anti-ERβ1, Anti-ERβ2, Anti-ERβ5 (Bio Rad, Hercules, CA, USA). Human anti-IL6 (GeneTex, San Antonio, Texas, USA). Human anti-GPER (Santa Cruz Biotechnology, CA, USA).

### Immunohistochemistry

Formalin-fixed, paraffin-embedded tissue blocks were retrieved from the archive and were analyzed by IHC described previously. [13, 24] In short, The Avidin-Biotin Complex (ABC) Vectastain Kit (ZSGB-Bio, Beijing, China) was used and the ERβ polyclonal antibody, IL6 polyclonal antibody, ERβ1 were used as primary antibodies incubated with tissue sections (4 mm) after heat-induced epitope retrieval (10 mM sodium citrate buffer of pH 6.0), followed by incubation with a secondary antibody conjugated to peroxidase (1:100; Dako). Detection was performed using diaminobenzidine for 3 min, and the slides were counterstained with hematoxylin. The scoring system that was used incorporated both the intensity of the scoring (0 = absent, −, 1 = weak, +, 2 = moderate, ++, 3 = strong, +++) and the percentage of positive tumor cells (0 = 0%, 1 = 1–25%, 2 = 26–50%, 3 = 51–75%, 4 = 76–100%). Points for the intensity and the percentage of staining were added and assigned as overall score according to the method described in Tang et al. [22]. Investigators scored all slides for ERβ, IL6 and ERβ1 staining and followed the criterions of double-blind trials. Based on the overall score, the ERβ, IL6 and ERβ1 staining results were classified into negative expression (≤4) and positive expression (> 4).

### Cells and transfection

Non-small cell lung cancer cells A549 and H1793 were obtained from the American Type Culture Collection (ATCC, Manassas, VA, USA). The normal human bronchial epithelial cell line HBE was obtained from Shanghai Cancer Institute. All cells were grown for 2 weeks (four passages) before freezing aliquots for subsequent analyses using the same batch of personally passaged cells. A549 and HBE Cells were propagated in RPMI 1640 medium (Gibco, Grand Island, NY, USA) supplemented with 10% fetal bovine serum (Gibco), and antibiotics (100 units/ml penicillin and 100 μg/ml streptomycin). While H1793 were cultured in DMEM: F12 medium (Boster, Wuhan, China) supplemented with 10% fetal bovine serum (Gibco), and antibiotics (100 units/ml penicillin and 100 μg/ml streptomycin). All cell lines were cultured at 37 °C in a humidified atmosphere of 5% CO2 and 95% air.

The expression vectors and Small interfering RNAs for ERβ were constructed as previously described [25]. Briefly, The ERβ-expressing plasmid and its empty plasmid were obtained from Origene Technologies Inc. (Rockville, USA). Small interfering RNAs targeting ERβ (ERβ-siRNA Stealth siRNAs HSS103378, Life Technologies, Carlsbad, CA) and control siRNAs (ctrl-siRNA, Life Technologies, Carlsbad, CA). Lipofectamine 3000 (Invitrogen, Life Technologies) were used during transfection following the manufacturer’s instructions. ShRNA-ERβ1, ShRNA-ERβ2 and ShRNA-ERβ5 shRNA lentiviral particles (GeneChem) were used to knock-down ShRNA-ERβ1/2/5 expression, targeting 5′- CCTTGCATGTGGGCAATGAAAGATT -3′, 5′- CGCTAAAGTGAAGACGGATTCTCTT -3′ and 5′- CACGTGCTTCGCGGGTGCAAGTCCT -3′ respectively. ShRNA-NC, the short hairpin RNA (shRNA) not targeting any known gene, was used as control. The cells with stable transfection were selected with puromycin.

### Drug treatment

The parental NSCLC cells or mouse model were treated with17β-estradiol, known as E2 (Sigma-Aldrich, St. Louis, MO, USA), Fulvestrant (Ful, an ER antagonist, Cayman Chemical, USA) either alone or as a combination. Recombinant human IL-6 (Cell Signaling Technology, Danvers, MA, USA), Stattic (Med Chem Express, USA) The dosages of these drugs used in vitro and vivo are comparable as previously described [20, 22] [25, 26]. Each group of cells was treated for 48 h and harvested for further analysis. Cell culture experiments were performed using reagents formulated in 100% DMSO.

### Western blotting of cultured cells and NSCLC samples

Western blot assay was performed following the protocol as previously described [25, 26]. Briefly, the cells were harvested and lysed using ice-cold RIPA lysis buffer (50 mM Tris-HCl (pH 7.4), 150 mM sodium chloride, 1% Nonidet P-40, and 0.5% sodium deoxycholate). NSCLC frozen tissues obtained from patients diagnosed with primary non-small cell lung cancer who underwent initial surgery at the Department of Thoracic Surgery, Affiliated Tongji Hospital of Huazhong University of Science and Technology Tongji Medical College (Wuhan, China). After unfreezed the frozen tissue and the exterior which is a white hard part was selected. Minced tissue lysed using ice-cold RIPA lysis buffer, homogenized the minced tissue use homogenizer (TIANGEN BIOTECH, China),

Following centrifugation at 10,000×g for 15 min at 4 °C, the proteins in the supernatants were quantified by Bradford method and separated using 10% SDS-PAGE gel and Electro-transferred from the gel to a nitrocellulose membrane (Merck & Co., Inc., Whitehouse Station, NJ, USA). Following blocking with 5% skimmed milk in phosphate-buffered saline, membranes cut up in stripes according to the different molecular weight. The membranes were immunoblotted with the primary antibodies, namely, IL6, ERβ, p-ERK, ERK, AKT, p-AKT, p-Stat3, Bcl-XL, GAPDH, ERβ1, ERβ2, ERβ5, GPER at 4 °C overnight. Specifically bound HRP-conjugated secondary antibodies were detectedusing an ECL detection system (ChemiDocTM XRS+ machine, Bio-Rad Laboratories). Protein levels of GAPDH were employed as loading controls. Densitometric analyses were performed using ImageJ software. Relative quantification was carried out after normalization to the band intensities of GAPDH. A Mann-Whitney test was performed to assess the difference of protein expression between groups. Each experiment was performed in triplicate and repeated at least 3 times.

### In vitro cell viability

Cell proliferation was evaluated using a colorimetric assay as described previously [26]. Cell viability was measured by a commercially Cell Counting Kit (CCK-8, Dojindo Molecular Technologies, Inc., Japan). The indicated cells were seeded into 96-well plates at a density of 10^4^ cells per well. The drugs were added when the plates were seeded. After incubation for 1, 2, 3 and 4 days, cell proliferation in 96-well plates was measured and 10ul CCK-8 reagent was added to each well and incubated at 37 °C for 1 h. Absorbance was measured at 450 nm by using a spectrophotometer. Each experiment was performed in triplicate and repeated at least 3 times.

### Wound healing, migration, and invasion assays

For the wound healing assay, transfected A549 and H1793 cells were seeded into 6-well plates and subjected to serum starvation for 24 h in serum-free media. Afterwards, an artificial wound was created in the confluent cell monolayer of cells. Photographs were taken at 0, 24, and 48 h using a scanner (Seiko Epson Corporation, Japan). Migration and invasion assays were conducted in Transwell chambers (Costar, Corning Inc., NY, USA) coated without or with Matrigel (BD Biosciences) on the upper surface of the 8-μm (pore size) membrane. Briefly, transfected A549 and H1793 cells were harvested, suspended in serum-free medium, and plated into the upper chamber for the migration or invasion assays, respectively, and media supplemented with 10% FBS were placed into the lower chamber. After 24 h incubation, the cells that had migrated or invaded through the membrane to the lower surface were fixed, stained, and counted under microscope (Olympus, Tokyo, Japan).

### Colony formation assay

The colony formation assay was performed on the indicated cells. For each group, 800 survived cells/well were incubated in 6-well plates, containing 2 ml complete medium per well, followed by incubation for 10 days. At the indicated time point, cells were washed twice with PBS, treated with crystal violet for 10 min, washed, then counted, and measured. All the experiments were performed at least 3 times. The colonies with > 40 cells were subsequently counted under a phase contrast microscope at × 40 magnification.

### Enzyme-linked immunosorbent assay

IL-6 protein levels were detected in culture medium using solid phase sandwich enzyme-linked immunosorbent assay (ELISA) assays according to the manufacturer’s protocol (D6050, R&D Systems). The IL-6 assay sensitivity was 0.7 pg/ml, and the assay range was 3.12–300 pg/ml. For the statistical analysis, culture medium was collected three times independently.

### Immunofluorescence staining of cultured cells and mouse lung sections

Cells cultured in cover slides were pretreated with drug stimulated or transfection for 48 h. The cells were fixed with 4% paraformaldehyde for 20 min, permeabilized with 0.1% Triton™ X-100 for 10 min, blocked with 5% BSA for 1 h and labeled with ERβ (1:100, Rabbit),IL6 (1:100, Mouse) at 4 °C overnight. Donkey anti-Rabbit and Donkey anti-Mouse IgG (H + L) Highly Cross-Adsorbed Secondary Antibody was used at a concentration of 2 μg/ml in phosphate buffered saline containing 0.2% BSA for 1 h at room temperature, and then counterstained with Hochest 33,342(1 μg/ml, 5 min, RT). Cells were visualized with fluorescence microscope (Olympus, Tokyo, Japan).

Mouse lung tumor were prepared and subjected to H&E staining as described previously [13]. The Avidin-Biotin Complex (ABC) Vectastain Kit (ZSGB-Bio, Beijing, China) was used and the ERβ (1:100, Rabbit),IL6 (1:100, Mouse) were used as primary antibodies incubated with tissue sections (4 mm) after heat-induced epitope retrieval (10 mM sodium citrate buffer of pH 6.0), and blocked with 5% BSA for 1 h. Donkey anti-Rabbit and Donkey anti-Mouse IgG (H + L) Highly Cross-Adsorbed Secondary Antibody was used at a concentration of 2 μg/ml in phosphate buffered saline containing 0.2% BSA for 1 h at room temperature, and then counterstained with Hochest. Tissue were visualized with fluorescence microscope (Olympus, Tokyo, Japan).

### Proteolytic degradation of collagen IV in the BM

Twenty four-well Matrigel invasion chambers were incubated with DQ-collagen IV (Invitrogen, Life Technologies) diluted in serum-free DMEM at a final concentration of 20 ng/ml at 37 °C in dark overnight. The chambers were briefly rinsed with serum-free DMEM. 1 × 10^4^cells were seeded into each invasion chamber in the presence or absence of pcDNA-ERβ and siRNA-ERβ. Cells were then incubated in a CO2 incubator at 37 °C for 4 h. Proteolytic degradation of DQ-collagen IV in the BM was imaged with fluorescence microscopy. When DQ-collagen IV was de-gradated by the MMPs secrete by cells, the clipped DQ-collagen may show fluorescence. A brighter fluorescence present the higher invasion ability. A study by Chen H et al. also described the protocol [27].

### Animal studies

#### Urethane-induced adenocarcinoma model and treatments

Four week old female Kunming mice (Animal Purchase No. 42009800000547) were obtained from the Experimental Animal Center of Hubei Province (Animal Study Permit No. SCXK 2010–0009) and maintained in an environment with a standardized barrier system (System Barrier Environment No.00021082) in the Experimental Animal Center of Tongji Hospital of Huazhong University of Science and Technology. After mice underwent ovariectomy to avoid the influence of endogenous estrogen, lung cancer was induced by urethane (Sigma-Aldrich, St.Louis, MO) as previously described [22]. When their vital signs remained stable, mice were divided into several groups (*n* = 6 per group): blank control, E2, E2 + Ful, the dosages of drugs as previously described [26]. After 14 weeks, mice were euthanized by cervical dislocation, and then, the lungs were collected. The number of tumor visible on the lungs was then determined.

#### The subcutaneous tumor model

An experimental model of A549 subcutaneous tumor model was used to investigate the effects of different ERβ isolation on the up-regulation of IL6 in vivo. A549 cell transfect ShRNA-NC, ShRNA-ERβ1, ShRNA-ERβ2 and ShRNA-ERβ5 shRNA lentiviral particles (GeneChem), cell visualized with fluorescence microscope detection the GFP fluorescence. Female Balb/c nude mice (Animal Purchase No.43004700006938) after mice underwent ovariectomy, A549 cells transfect ShRNA-NC, ShRNA-ERβ1, ShRNA-ERβ2 and ShRNA-ERβ5 shRNA lentiviral particles respectively (5 × 106/100ul) suspended in PBS were injected into 4-week-old female BALB/c nude mice subcutaneous. Drugs were subcutaneously injected twice weekly in a volume of 100ul per mouse, respectively. After 4 weeks of treatment, mice were euthanized by cervical dislocation. The subcutaneous tumor were collected.

### Bioinformatics analysis

The prognostic value of the IL6/ERβ was analyzed by a Web-based Kaplan–Meier plotter (http://www.kmplot.com/lung), which is a meta-analysis tool of gene expression and survival data of 2437 lung cancer patients (2015 version) using multiple microarray data [28, 29].

### Statistical analysis

In general, unpaired two-tailed Student *t* test and one-way ANOVA were used to make inter-group comparison. The Kaplan-Meier method was used to estimate overall survival. The univariate and multivariate Cox proportional hazards modeling was used to evaluate prognostic significance. For cell-based assays, differences between groups were assessed by two-tailed Student t tests, unless indicated otherwise. Data were analyzed using SPSS 18.0 statistic software (SPSS Inc., Chicago, IL). Each experiment was repeated at least 3 times with comparable results, unless indicated otherwise. All results were presented as mean ± SD (standard deviation) with a *P* value < 0.05 considered statistically significant.

## Result

### IL6 and ERβ are upregulated in lung cancer and are associated with poor prognosis

**Fig. 1:**
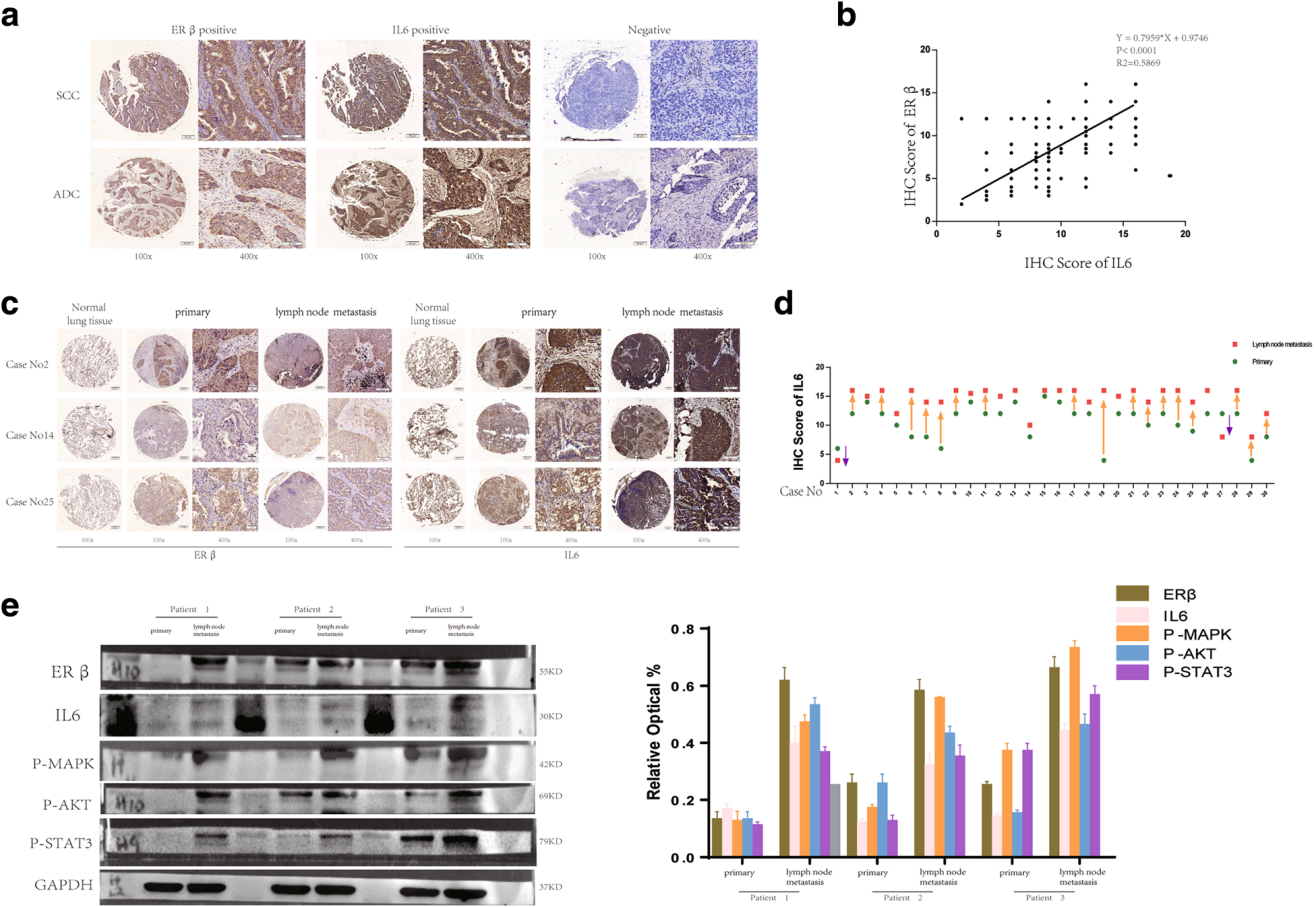
IL6 is upregulated in lung cancer and correlates with ERβ expression. (**a**) Immunohistochemical staining of IL6 and ERβ protein in human lung cancer tissue. The brown color in cancer cells denotes positive staining. A negative control was performed without primary antibody. Representative images of IL6 and ERβ expression in NSCLC are shown, including both squamous carcinoma and adenocarcinoma. Original magnification × 100 and × 400. Scale bars 50 and 20 μm respectively. (**b**) The relative level of IL6 expression was plotted against the relative level of ERβ expression in 289 samples (r (spearman) =0.263; *p* = 0.004). (**c**) Primary NSCLC tumor tissues and metastatic lymph node tissues in three typical cases (original magnification 100× (left) and 400× (right)). (**d**) IHC scores of 30 pairs of specimens from both primary tumor tissue and metastatic lymph nodes (□: lymph nodes metastasis; ○: primary tumor). (**e**) Western blotting analysis of IL6 expression in three paired primary NSCLC tissues and matched metastatic lymph node tissues. GAPDH was used as a loading control

In our previous study, the expression of ERβ in NSCLC tissues and corresponding normal lung tissues of 60 patients was reported [24], and we confirmed the overexpression of ERβ in NSCLC. IL6 has been similarly reported to be overexpressed in lung cancer [30, 31]. To screen for expression of IL6 and ERβ in NSCLC, 289 NSCLC samples were collected. Immunohistochemical staining for IL6 and ERβ expression was performed on both cancer tissues and adjacent non-cancerous tissues from 289 patients. The results indicate that IL6 and ERβ are absent in pneumocytes from normal lung tissues (Fig. 1c, Additional file 1: Figure S1A), but both of them were significantly increased in NSCLC (Fig. 1a). Positive ERβ expression was observed in 89.27% of specimens (258 / 289), while positive IL6 expression was observed in 93.43% (270 / 289). Next, we analyzed the relationship between ERβ/IL6 expression levels and clinicopathological characteristics. As shown in Table 1, no statistically significant correlations were observed between expression of ERβ/IL6 and gender, age, lymph node metastasis, TNM stage, or tumor histology at diagnosis (*p* > 0.05). However, statistically significant correlations for high levels of ERβ/IL6 expression were found with clinically distant metastasis and poor differentiation levels (*p* < 0.05). Additionally, smoking was associated with IL6 expression (*p* = 0.022) but not with ERβ expression (*p* = 0.227). When we grouped samples according to their distinct IHC scores by tumor differentiation and metastasis status, those with poor differentiation or metastasis harbored higher ERβ/IL6 expression (Additional file 1: Figure S1B, C). In addition, statistical analysis showed a Spearman correlation coefficient of 0.263 (*p* = 0.004) (Table 2). The relative level of IL6 expression was plotted against the relative level of ERβ expression in these samples, suggesting a significant positive linear correlation between ERβ and IL6 expression (Fig. 1b) in NSCLC.

**Table 2:** The relationship between ERβ and IL6 expression levels of 289 NSCLC tissue

	Expression	ERβ			
		Positive	Negative	*P*-value	Sperman’s rho
IL6	Positive	254	16	0.004	0.263
	Negative	4	15		

Furthermore, the association between high ERβ/IL6 mRNA expression and poor prognosis in NSCLC was analyzed using the online Kaplan–Meier survival analysis of expression data (probe210780/ 205207_x_at) from 1928 NSCLC patients (http://www.kmplot.com/lung). The results show that patients with high ERβ/IL6 expression exhibited shorter overall survival compared to patients with low ERβ/IL6 expression (*P* < 0.05, log-rank test). Interestingly, we found that although IHC detection of IL6 expression showed no significant correlations with tumor histology, the overall survival of differing histology was indeed associated with IL6 expression. Overall survival was shorter in patients exhibiting high expression of IL6 than in those showing low expression in the lung adenocarcinoma group (*P* < 0.001, log-rank test), but not in the lung squamous cell carcinoma group (*P* = 0.3175, log-rank test) (Additional file 1: Figure S1D).

Taken together, these results suggest that both IL6 and ERβ expression are significantly increased in malignant NSCLC. There is a correlation between high expression of IL6 and ERβ. Higher ERβ/IL6 expression is also associated with poor histopathological grade and metastasis in NSCLC patients. Importantly, ERβ/IL6 expression was identified as an independent prognostic factor for NSCLC, with higher IL6 expression indicating a shorter overall survival in lung adenocarcinoma.

### IL6 expression is significantly higher in metastatic lymph nodes than in primary NSCLC tumor tissue

In Table 1, we show that IL6 and ERβ are correlated with clinically distant metastasis. To elucidate the relationship between ERβ/IL6 expression in primary tumors and paired metastatic lymph nodes, we evaluated 60 samples in the paired primary tumors and metastatic lymph nodes using IHC analysis, which we described before [25]. In our previous study, we found that ERβ was more highly expressed in the metastatic than in the primary tumors [25]. The results of the IHC analysis are summarized in Table 3. The paired samples t-test shows that ERβ/IL6 expression in metastatic lymph nodes was significantly higher than that in primary tumor tissue (*p* < 0.001, Table 3). Likewise, compared to paired metastatic lymph nodes, the primary tumors exhibited lower ERβ/IL6 expression (Fig. 1c). When comparing IL6 IHC scores between primary tumor and metastatic lymph nodes, we see a rising trend (Fig. 1d). These results are consistent with our previous study [25]. Additionally, three NSCLC patients were chosen who had surgery at the Department of Thoracic Surgery, Affiliated Tongji Hospital of Huazhong University of Science and Technology Tongji Medical College (Wuhan, China) whose postoperative pathological diagnosis was revealed as NSCLC. We collected both primary tumor tissue and metastatic lymph node tissue and measured ERβ/IL6 protein expression. Similarly, our results showed that both ERβ and IL6 were overexpressed in NSCLC metastatic lymph nodes rather than in the primary tumor (Fig. 1e). This suggests a marked difference in the activation of the ERK/AKT/Stat signal transduction pathway between these samples.

**Table 3:** List of biopsies included in this study with patient clinicopathologic parameters and immunohistochemical staining scores for ERβ and IL6, the relationship of ERβ/IL6 expression lever between primary tumor and metastatic lymph nodes

Case No.	Gender	Age at time of surgery	Tumor Stage UICC	Tumor Stage TNM	Lymphnode metastasis ratio	IHC score of ERβ		IHC score of IL6	
						Primary Tumor	Lymphnode Metastasis	Primary Tumor	Lymphnode Metastasis
01	Female	48	IIIA	pT2aN2	11/13	++	++	+	–
02	Male	64	IIIA	pT2aN2	11/26	+	+++	++	+++
03	Male	66	IIIA	pT2bN2	5/21	++	+++	++	+++
04	Male	21	IIIA	pT2bN2	6/26	++	++	+++	+++
05	Male	63	IIIA	pT2bN2	4/16	++	++	++	++
06	Male	57	IIIA	pT2aN2	7/16	+	+++	++	+++
07	Male	46	IIIA	pT2aN2	5/27	+++	+++	++	++
08	Female	58	IIIB	pT3N3	4/14	+	+++	+	++
09	Male	52	IIIA	pT2aN2	5/18	++	++	++	+++
10	Male	38	IIIB	pT4N2	4/16	++	++	++	++
11	Male	60	IIB	pT2bN1	2/40	+	+++	++	+++
12	Male	58	IIIA	pT3N2	11/18	+++	+++	++	+++
13	Male	53	IIIB	pT2bN3	14/20	++	+++	++	+++
14	Female	57	IIIB	pT1bN3	9/12	+++	+++	++	+++
15	Male	42	IIIA	pT2aN2	14/32	+++	+++	++	++
16	Male	72	IIIA	pT2aN2	7/11	++	+++	+	+
17	Male	64	IIB	pT2aN1	1/10	++	++	+	+++
18	Male	61	IIIA	pT2bN2	2/10	+++	+++	+++	+++
19	Male	65	IIIB	pT4N2	1/32	+++	+++	–	+++
20	Male	66	IIIA	pT2bN2	4/8	+++	++	++	++
21	Male	67	IIB	pT2aN1	2/14	++	+++	++	++
22	Male	53	IIIA	pT2aN2	11/23	+++	+++	++	+++
23	Male	59	IIIA	pT2aN2	16/31	++	+++	++	++
24	Male	45	IIIB	pT2aN3	8/11	+	+++	++	+++
25	Male	60	IIIA	pT3N2	9/35	+++	++	+	++
26	Male	59	IIIA	pT2aN2	7/20	++	+++	+++	+++
27	Male	47	IIIA	pT2aN2	12/18	++	+++	++	+
28	Male	69	IIIA	pT2bN2	2/37	+	+++	+++	++
29	Male	57	IIIA	pT3N1	1/11	+	+++	++	+
30	Male	60	IIIA	pT2aN2	5/11	++	+++	++	++
primary vs.LN						Median score	Median score	Median score	Median score
			<0.001			11.10	14.57	11.29	13.58
						*p* value	<0.001	*p* value	<0.001

In summary, all these data suggest that ERβ/IL6 expression is significantly higher in metastatic lymph nodes than in primary NSCLC tumor tissue. Expression of ERβ and IL6 may play an important role in NSCLC metastasis.

### E2 positively regulates IL6 expression and activates PI3K/AKT and MAPK/ERK pathways

**Fig. 2:**
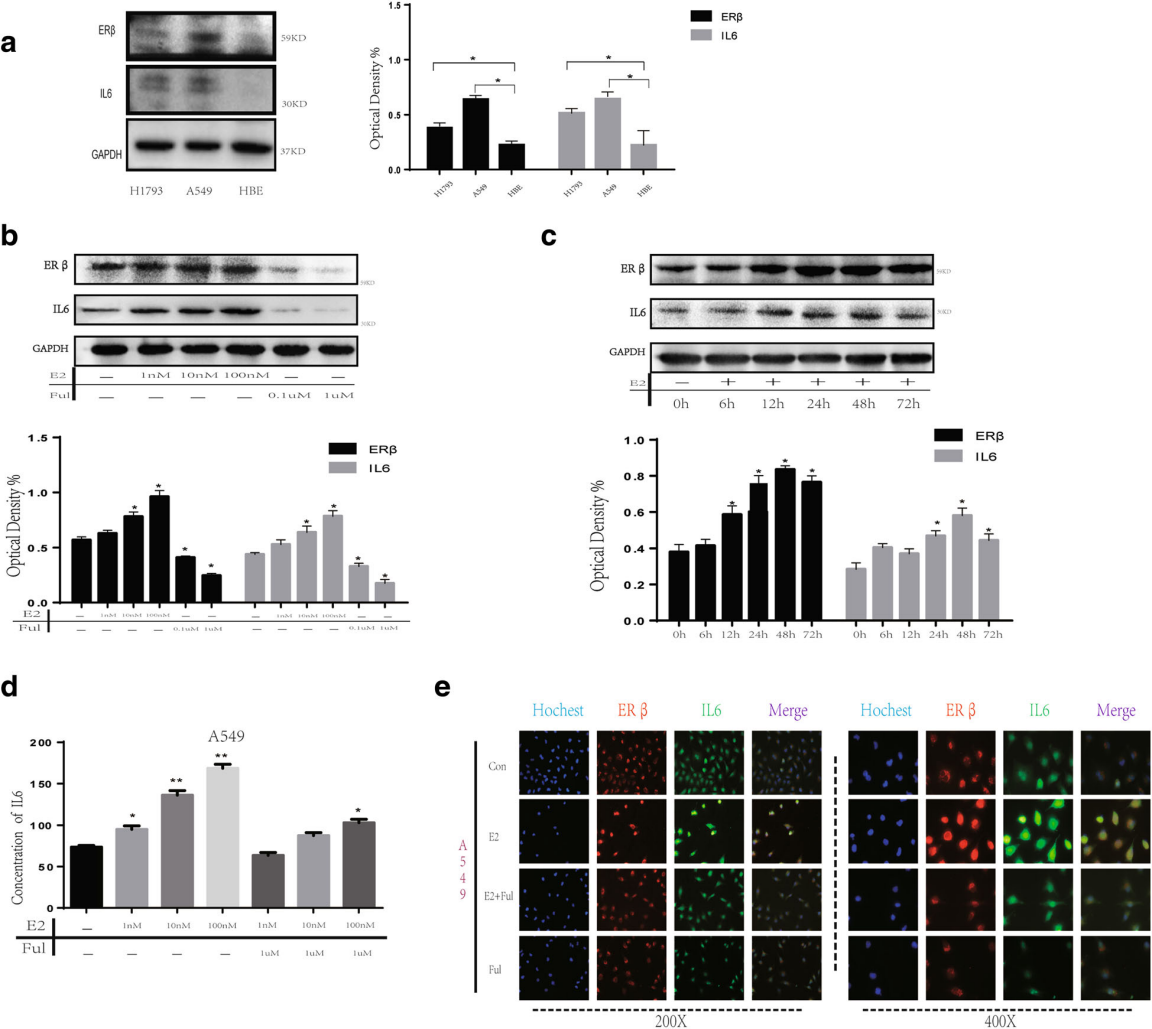
Upregulation of IL6 stimulated by E2 in NSCLC cell lines, A549 and H975. (**a**) ERβ/IL6 expression was up regulated in NSCLC cell lines compared with normal pneumocytes (HBE). (**b**) Synchronized cells were treated with E2 at different concentrations (0 nM, 1 nM, 10 nM and 100 nM) and Ful (0.1 μM and 1 μM) for 2 days. The protein expression of ERβ and IL6 was analyzed using western blot. (**c**) Synchronized cells were treated with E2 at different time points (0 h, 6 h, 12 h, 24 h, 48 h and 72 h) at concentrations of 10 nM. The protein expression of ERβ and IL6 was analyzed using western blot. GAPDH was used as a control. (**d**) Autocrine IL6 was analyzed by ELISA assay after concentration-dependent treatment of E2 (10 nM) or Ful (0.1uM). (**e**) The upregulation of IL6 in A549 after stimulation with E2 (10 nM) or Ful (0.1uM) was determined by immunofluorescence

Given that IL6 over expression is strongly correlated with malignant behavior in NSCLC, especially in lung adenocarcinoma (Additional file 1: Figure S1D), the expression of ERβ and IL6 in a normal bronchial epithelial cell line (HBE) and lung adenocarcinoma cell lines A549 and H1793 was evaluated by western blot. For both A549 and H1793 was commonly used to explore the relationship between estrogen and lung cancer, also our previously studies confirmed A549 and H1793 have good estrogen reactivity. ERβ and IL6 expression was markedly higher in NSCLC cell lines than in normal lung epithelial cells (Fig. 2a). In addition, ERβ/IL6 expression in cell lines coincided with the results in patient tissue.

17β-estradiol (E2) is an ER selective agonist and fulvestrant (Ful) an ER antagonist that inhibits ERβ activation by E2. In this study, we performed western blots to further study stimulation of IL6 by 17β-estradiol through activated ERβ in NSCLC cell lines. The results show that expression of ERβ was significantly increased after E2 treatment in a dose- and time-dependent manner, as we previously reported [25]. Interestingly, we observed similar dose- and time-dependent expression of IL6 upon stimulation with E2 (Fig. 2b, c). Next, we examined A549 and H1793 cell growth in response to E2 (10 nM), E2 + Ful and Ful (1 μM) administration, analyzed by western blot and ELISA after two days of treatment. As expected, IL6 was induced when ERβ was activated, while when co-treated or treated with Ful alone, IL6 expression was inhibited (Fig. 2d; Additional file 2: Figure S2A). Similarly, in A549 and H1793 cells, ERβ activated by E2 also enhanced IL6 expression, as shown by ELISA and immunofluorescence (IF) (Fig. 2e; Additional file 2: Figure S2A, B, F). Taken together, these results clearly indicate that E2 stimulates IL6 expression.

**Fig. 3:**
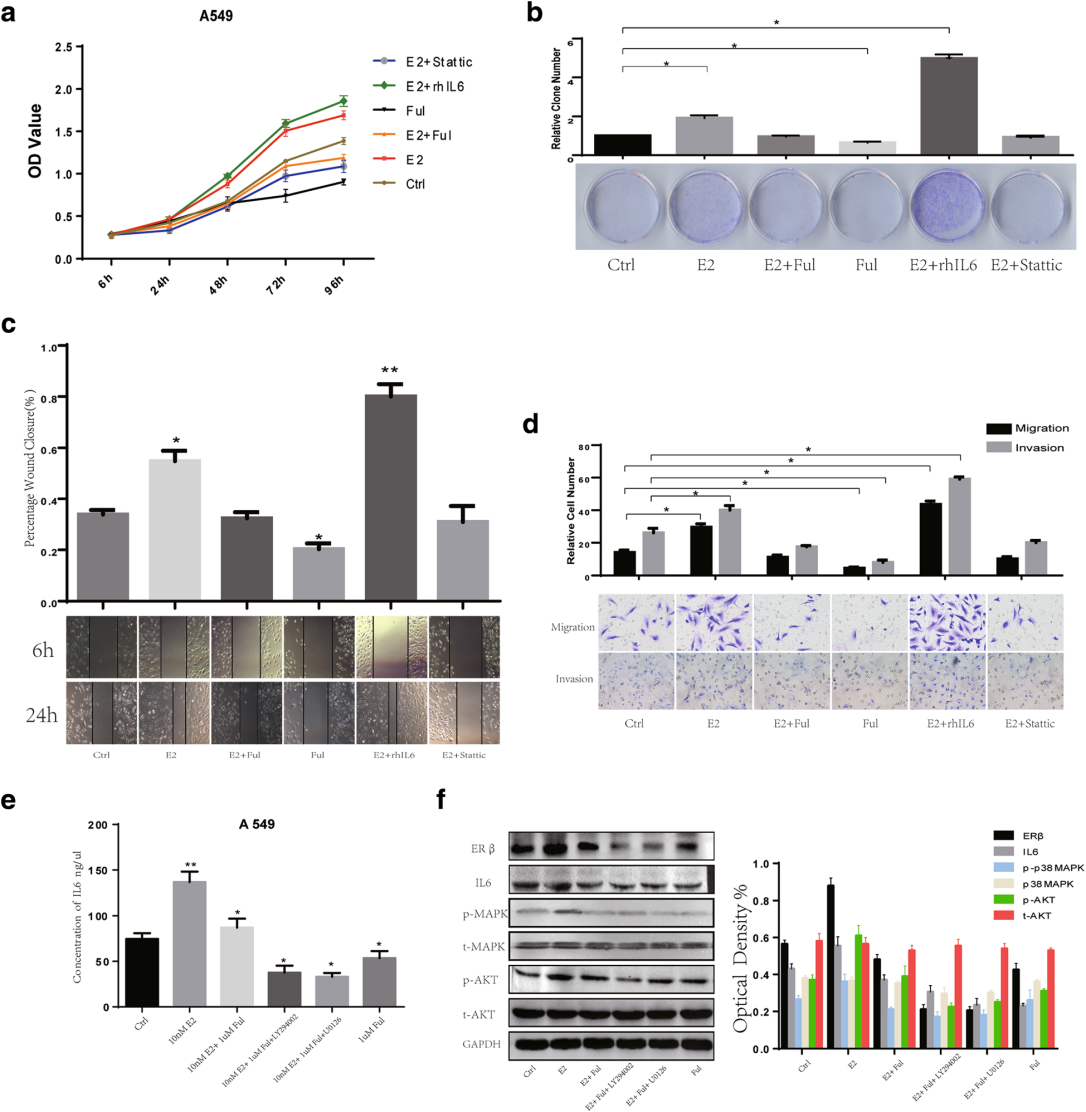
Upregulation of IL6 stimulated by E2 treatment regulates aggressiveness of NSCLC cells. (**a**) The cell proliferation of A549 was determined by CCK-8 assay after 72 h concentration-dependent treatment of E2 (10 nM) or Ful (0.1uM) or rhIL6 (0.5 ng/ml) or Stat3 inhibitor static (20uM). The results are representative of three independent experiments. (**b**) Colony formation assay measuring the proliferative activity in A549 cells after concentration-dependent treatment of E2 (10 nM) or Ful (0.1uM) or rhIL6 (0.5 ng/ml) or Stat3 inhibitor static (20uM). (**c**) Wound-healing assays were performed to assess NSCLC cell migration. Wound closure was determined 24 h after the scratch. (**d**) Transwell assay was used to quantify cell migration and invasion capacity. The average number of cells per field of view in three different experiments is plotted. (**e**-**f**) ELISA and western blot were used to detect the effect of E2 (10 nM) and its receptor antagonist Ful (0.1uM) on IL6 expression and the influence of MEK inhibitor U0126 (60 nM) or a selective PI3K inhibitor of LY294002 (0.6uM) on E2-mediated IL6 expression through MEK/ERK and PI3K/AKT activation in A549 cells

Considering that ER signaling in cancer requires activation of downstream PI3K/AKT and MAPK/ERK pathways [12], E2 could regulate IL6 expression and phosphorylation of p38MAPK and AKT, which were inhibited by Ful treatment. To test this, we used LY294002 (0.6 μM, a selective inhibitor of PI3K) and U0126 (60 nM, a selective inhibitor of MEK) for subsequent experiments. Our results indicate that administration of LY294002 or Y0126 sufficiently blocked the enhancement effect of E2 on ERβ, as well as IL6, expression and AKT/ERK activity (Fig. 3e, f; Additional file 2: Figure S2F).

Collectively, these data suggest that E2 regulates IL6 expression via activation of PI3K/AKT and MAPK/ERK pathways.

### ERβ and IL6 up-regulated by E2 enhance aggressiveness of lung cancer cells

E2 positively regulates IL6 and ERβ expression; however, whether IL6 and ERβ expression stimulated by E2 enhances the aggressiveness of NSCLC cells is unknown. This experiment was divided into groups administered with E2, E2 + Ful, Ful, rhIL6 and Stattic (20uM, a selective inhibitor of Stat3 which is the major signal pathway of IL6).

To determine if E2 treatment leads to an increase in the proliferation and malignancy of NSCLC cells, CCK8, wound healing, Transwell and colony formation assays were performed. The CCK8 assay showed that cell growth was significantly increased by E2 treatment or co-treatment with rhIL6 and decreased by Ful treatment or co-treatment with Stattic in A549 cells at 48/72/96 h (*P* < 0.05, Fig. 3a). The colony formation of A549 and H1793 cells treated with E2, Ful and rhIL6 single agents or in combination was also significantly different between groups. The colony formation ability of cells treated with E2 or co-treated with rhIL6 was significantly higher than those treated with Ful or co-treatment with Stattic (all *P* < 0.05, Fig. 3b; Additional file 2: Figure S2C). The wound healing assay demonstrated that the migratory ability of A549 and H1793 cells treated with E2 was significantly higher than that of cells receiving Ful, co-treatment with rhIL6 enhance the migratory ability while co-treatment with Stattic reverse the effect (Fig. 3c; Additional file 2: Figure S2D). Similarly, the Transwell migration assay showed that treatment with E2 significantly increased the migratory ability of NSCLC cells, while Ful treatment showed the opposite (*P* < 0.05). Moreover, the Transwell invasion assay revealed that E2 treatment significantly enhanced the invasiveness of NSCLC cells compared to cells treated with Ful (*P* < 0.05). Meanwhile co-treatment with rhIL6 can enhance the effect induced by E2, treatment with Stattic can reverse both the migratory and invasion ability (*P* < 0.05, Fig. 3d; Additional file 2: Figure S2E).

These results indicate that E2 stimulation significantly promotes aggressiveness of lung cancer cells in vitro. Considering both IL6 and aggressiveness of lung cancer cells up-regulation upon treatment with E2, meanwhile blocking IL6/Stat3 signal pathway reverse the effect. E2 may promote IL6 expression and synergy in NSCLC tumors.

### E2 regulates IL6 expression through ERβ and increases the malignancy of lung cancer cells

**Fig. 4:**
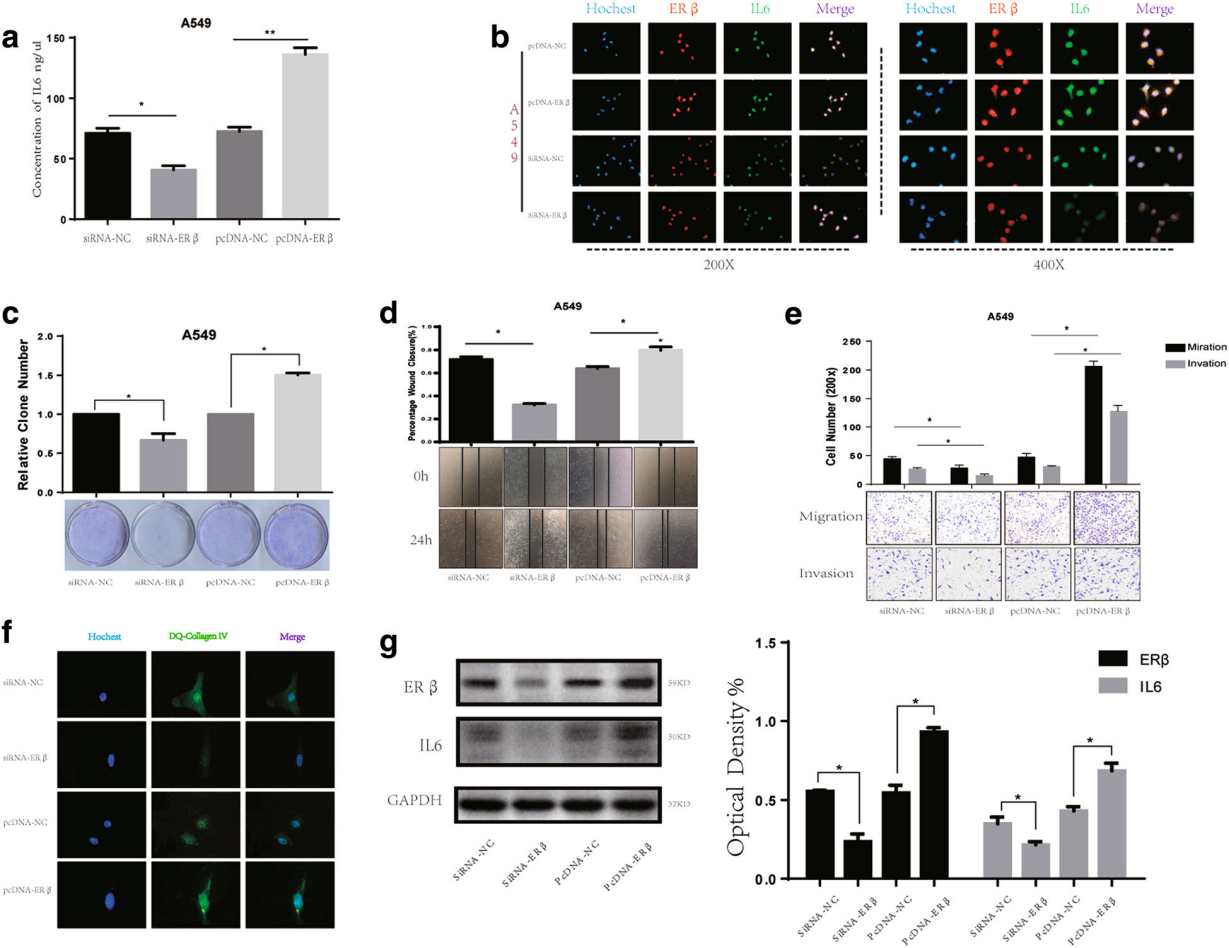
E2 regulates IL6 expression through ERβ and affects malignancy of lung cancer. (**a**) Autocrine IL6 was analyzed by ELISA assay after overexpression or knockdown of ERβ. (**b**) Upregulation of IL6 induced by E2 (10 nM) treatment 48 h was determined by immunofluorescence in A549. (**c**) Colony formation assay measuring the proliferative activity in A549 cells after overexpression or knockdown of ERβ. (**d**) Wound-healing assays were performed to assess NSCLC cell migration in response to changes in ERβ expression. (**e**-**f**) Transwell assay and DQ-collagen invasion assay were used to quantify the effect of ERβ regulation on cell migration and invasion capacity. (**g**) Expression of IL6 by western blotting after modulation of ERβ expression

To further explore whether ectopic expression of ERβ affects IL6 expression and aggressiveness of lung cancer cells, we chose two cell lines, A549 and H1793, to knockdown and overexpress ERβ. The effects of siRNA transfection and plasmid transfection on ERβ expression were confirmed by western blot, ELISA and immunofluorescence. IL6 expression in A549 and H1793 cells overexpressing ERβ was significantly higher than that in cells transfected with empty vector. Likewise, knockdown of ERβ expression with siRNA led to a decrease in IL6 expression (Fig. 4a, b, g; Additional file 3: Figure S3A, B).

To determine if ERβ affects malignancy in NSCLC cells, wound healing, Transwell and colony formation assays were performed. As expected, the wound healing and colony formation assays demonstrated significantly higher migratory abilities of A549 and H1793 cells with ERβ overexpression compared to cells transfected with empty vector. On the contrary, ERβ silenced cells showed significantly decreased migration (*P* < 0.05) compared to cells in the control groups (Fig. 4c, d; Additional file 3: Figure S3C, D). Similar results were obtained in Transwell and DQ-collagen invasion assays, with ERβ-overexpressing A549 and H1793 cells significantly promoting cell migration and invasion compared with the pcDNA3.1-control group. Likewise, silencing ERβ reversed these effects (*p* < 0.05, Fig. 4e, f; Additional file 4: Figure S3E).

Our data indicate that ERβ expression is essential for expression of IL6 stimulated by E2. In addition, ERβ expression affects the aggressive of lung cancer cells via E2 regulation of IL6 expression. Thus, these data strongly suggest that E2 regulates IL6 expression through activation of ERβ to regulate lung cancer cell metastasis.

### Overexpression of IL6 stimulated by E2 promotes growth of NSCLC xenograft tumors in vivo

**Fig. 5:**
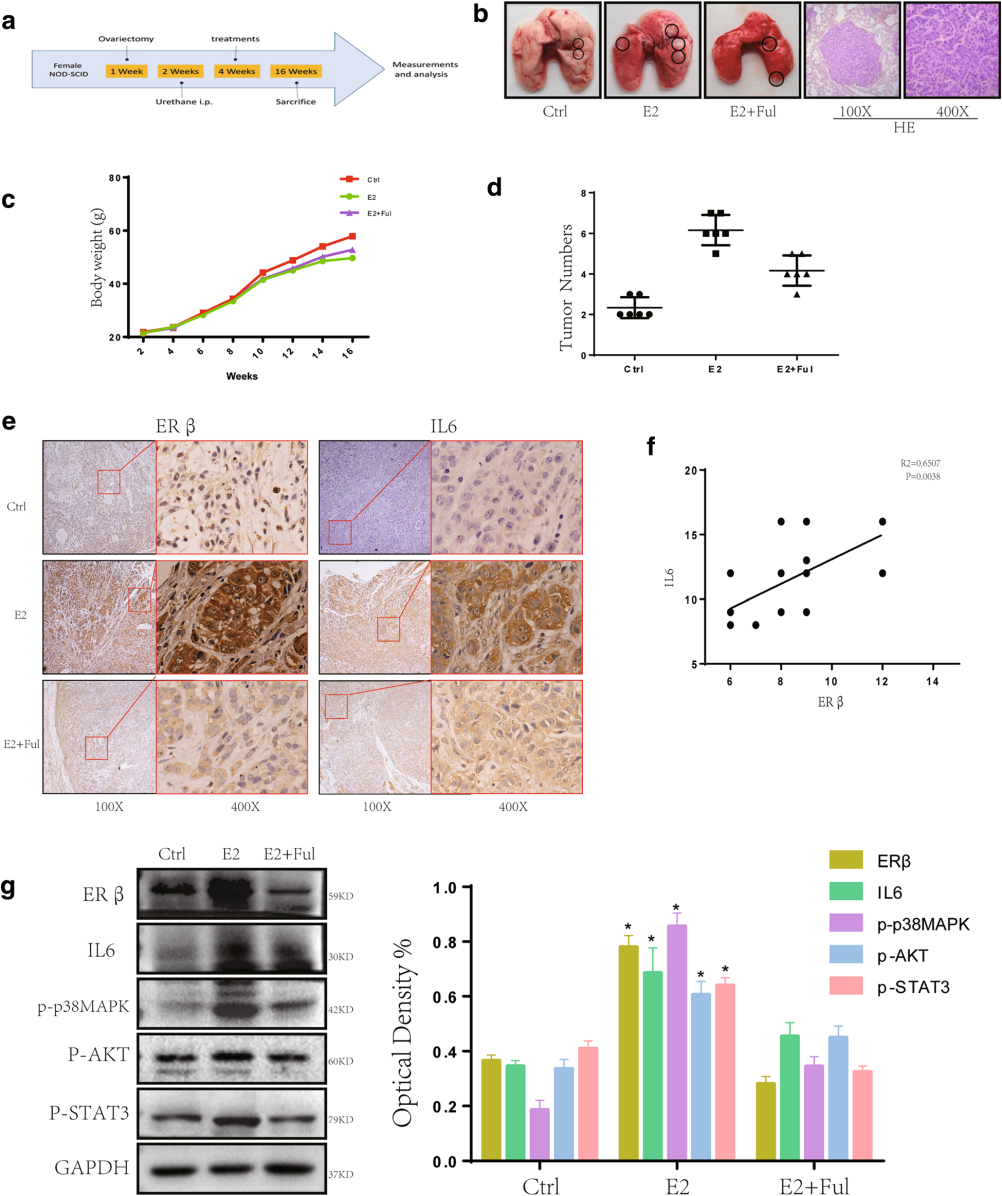
Upregulation of IL6 stimulated by E2 induces tumorigenesis in a urethane-induced adenocarcinoma model. (**a**) The process of creating a urethane-induced adenocarcinoma model illustrated in a simplified sequence flow diagram. (**b**-**d**) Two weeks after urethane was injected, mice were randomly divided into 3 groups (*N* = 6/group): E2 (0.09 mg/kg), E2+ Ful (1.46 mg/kg) and blank control. Lungs were removed after 12 weeks of subcutaneous drug treatment. The gross appearance of lung tumor nodes in different groups is indicated by black circles. The tumor numbers of different groups and tumor growth curves were obtained. (**e**-**f**) IHC staining of ERβ and IL6 shows a significant positive linear correlation between ERβ and IL6 expression. (**g**) Protein expression of ERβ, IL6, p-p38MAPK, p-AKT and p-Stat3 in murine lung tumors was analyzed using western blot. All data are expressed as the mean ± SD. Student’s t-test was used for statistical analysis

To further explore whether ectopic expression of IL6 stimulated by E2 affects tumor growth in vivo, we established a urethane-induced adenocarcinoma model (Fig. 5a). After 12 weeks of treatment, mice from each group (*n* = 6) were sacrificed, and visible tumors were observed on lung surfaces. The number of tumors observed in the E2 group was significantly greater than that in the control group. However, co-treatment with Ful reversed this phenomenon (Fig. 5b, d). We next performed IHC and IF analysis for ERβ and IL6 on these tumor masses. ERβ, as well as IL6, was significantly increased in E2-treated groups compared to untreated groups or co-treatment groups (Fig. 5e; Additional file 4: Figure S4A). When we plotted the relative level of IL6 expression against the relative level of ERβ expression in tumor samples, we observed a significant positive linear correlation between ERβ and IL6 expression (R^2^ = 0.6507, *P* = 0.003, Fig. 5f).

We next used western blot to measure the expression of ERβ and IL6 in mouse tumor tissue. The expression of ERβ and IL6 in the E2 group was significantly higher than that in the control group, while being decreased in the E2 + Ful group. Compared with the control group, we also found increased p-p38MAPK, p-AKT and p-Stat3 levels following stimulation by E2 (Fig. 5g).

Taken together, these results demonstrate that E2 up-regulate IL6 expression by activating ERβ via activation of PI3K/AKT and MAPK/ERK pathways to promote tumor growth in vivo.

### ERβ subtypes play differential roles in E2 stimulation of IL6 expression

In our previous study, we confirmed that only ERβ1, 2 and 5 expression was observed in human NSCLC tissue, suggesting that the different isoforms may play differential roles in lung cancer [13]. Given these observations, we utilized an experimental model of an A549 subcutaneous tumor model to investigate the effects of different ERβ isoforms on the up-regulation of IL6 in vivo. We transfected shRNA-NC, shRNA-ERβ1, shRNA-ERβ2 and shRNA-ERβ5 shRNA lentiviral particles (GeneChem) in A549 cells and performed imaging with fluorescence microscopy to detect GFP fluorescence and western blot analysis to verify transfection efficiency (Additional file 4: Figure S4B, C).

**Fig. 6:**
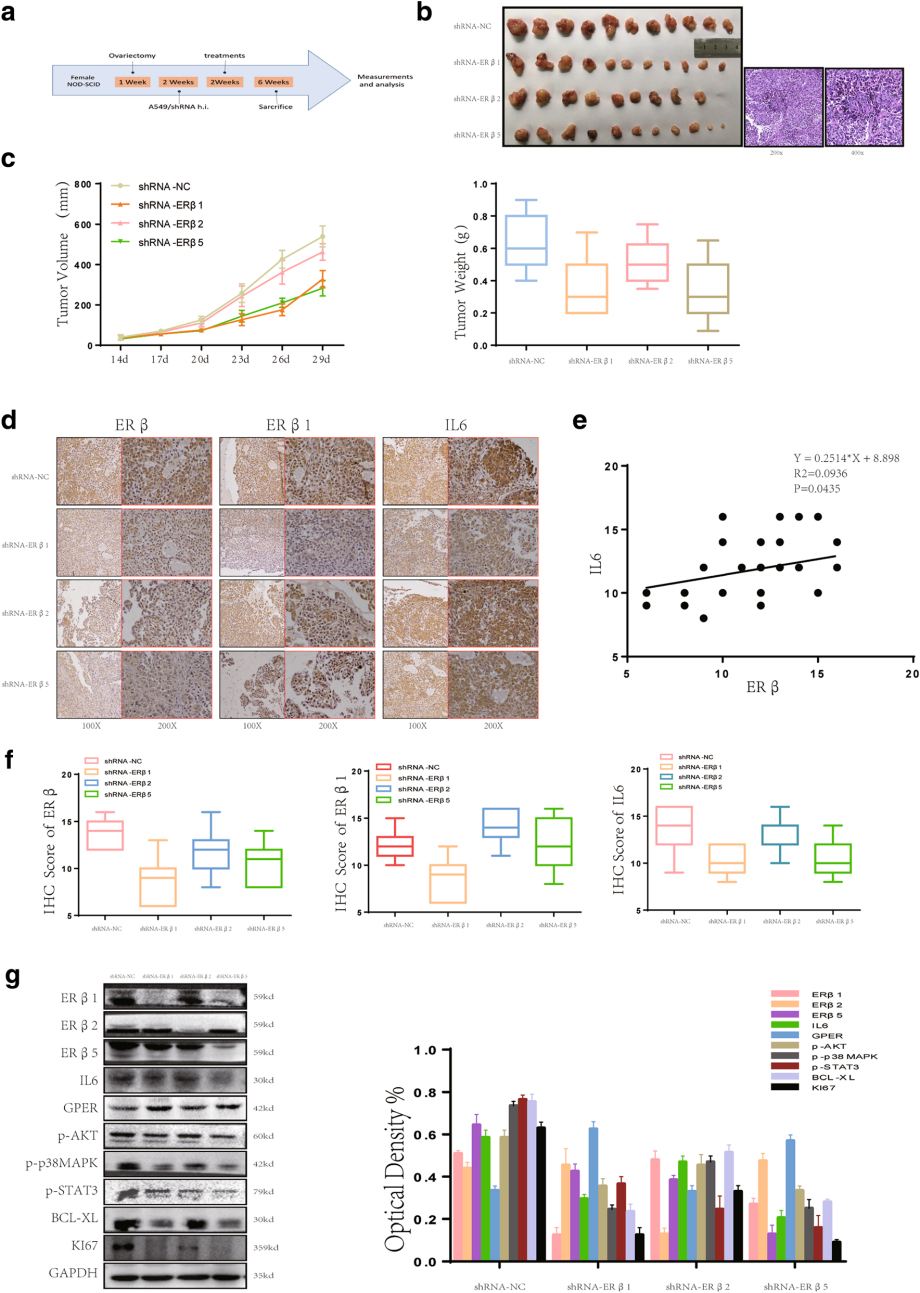
ERβ isoforms play differential roles in E2-stimulated IL6 expression. (**a**) The creation of our xenograft mouse model illustrated in a simplified sequence flow diagram. (**b**-**c**) Ovariectomized mice were subcutaneously injected with A549 cells transfected with shRNA-NC, shRNA-ERβ1, shRNA-ERβ2 or shRNA-ERβ5 shRNA lentiviral particles (GeneChem) (*N* = 8/group). Mice were euthanized and photographed after 4 weeks of E2 (0.09 mg/kg) treatment. The tumor photograph, tumor weight and tumor growth curves of each group were obtained. (**d**-**f**) IHC staining of ERβ, ERβ1 and IL6 showed a significant positive linear correlation between ERβ and IL6 expression. All data are presented as the mean of three independent experiments ± SD. (**g**) The protein expression of ERβ subtypes, IL6, p-p38MAPK, p-AKT and p-Stat3 in murine lung tumors was measured using western blot. All data are expressed as the mean ± SD. Student’s t-test was performed to assess statistical significance

We created a xenograft tumor model by subcutaneously injecting A549 cells with ERβ1, 2 and 5 knockdown or cells transfected with empty vector into the front flank of BALB/c nude mice (*N* = 8/group) (Fig. 6a). The results showed that tumors injected with ERβ1 or 5 knockdown cells grew more slowly and were smaller compared with tumors injected with cells containing empty vector or shRNA-ERβ2 (*P* < 0.05, Fig. 6b, c). Furthermore, IHC analysis revealed that ERβ1 or 5 knockdown reversed this effect, while ERβ2 knockdown did not (Fig. 6d, f). Analysis of the correlation between IL6 and ERβ IHC score in different ER subtype knockdown tumors showed that a significant positive linear correlation existed between ERβ and IL6 expression (R^2^ = 0.0936, *P* = 0.043, Fig. 6e). Next, we determined the expression of IL6, ERβ, ERβ isolation and other proliferation and apoptosis related proteins by western blot. ERβ1 or 5 knockdown tumors exhibited lower IL6 expression along with decreased expression of p-AKT, p-p38MAPK, Ki67 and BCL-XL compared with the NC or ERβ2 knockdown groups (Fig. 6g).

These data indicate that ERβ1 and 5 isoforms play a key role in the process of E2-stimulated IL6 expression and promote the growth of NSCLC xenograft tumors in vivo.

## Discussion

Increasing lines of evidence have confirmed that estrogen is directly correlated with NSCLC. Aromatase, which is essential to estrogen synthesis in tumors, is highly expressed in lung tumor tissue [5, 32, 33]. Preclinical data have confirmed that estrogen induces lung cancer [34]. ERβ is the dominant ER stimulated by E2 in the development of NSCLC [9, 10, 35]. ERβ is expressed as five protein isoforms, but only ERβ1, ERβ2 and ERβ5 expression has been documented in NSCLC [6, 13, 36, 37]. Transcription factors regulate gene transcription by binding directly or indirectly to the estrogen response elements (EREs) in the enhancer and/or promoter of target genes [38]; however, in the cytosol, ERs can active Src kinase, MAPK and AKT pathways, processes known as rapid signaling [39]. In fact, the AF-1 (activating function-1) domain in ERβ is less functional with regard to transcriptional activation compared to AF-1 in ERα [7, 40]. These differences in functional homology between ERα and ERβ may partially account for the observed differences in E2 responsiveness. The previous study revealed that estrogen induces cell proliferation of NSCLC cells through ERs in vitro and in vivo*, however* compared with ERα, non-genetic signaling to ERβ may play a major role in lung cancer [10, 11, 36, 41]. In our study the result confirm that ERβ play a vital role in lung cancer. Meanwhile, IL6 is associated with cancer development, including lung cancer [15, 16, 42]. Ectopic expression of IL-6 is correlated with tumor progression and overall survival in patients with NSCLC [15, 18]. Blockade of IL6 suppresses proliferation of lung cancer stem cells [43]. Higher levels of postoperative serum IL6 indicate higher risk of early postoperative recurrence [19]. Moreover, an extensive body of research has demonstrated that the downstream signaling of IL6 and that of ERβ do indeed intersect [21, 44, 45].

Taken together, both ERβ and IL6 are important in the pathogenesis of lung adenocarcinoma. Wang et al. reported that Stat3, a downstream signaling molecules of IL6, can up-regulate expression of ERβ [15, 21]. However, whether E2 stimulation can mediate IL6 expression in lung cancer was unclear. In this study, we presented evidence that IL6 is a downstream target gene of ERβ. To identify the relationship between IL6 and ERβ in NSCLC, we performed IHC and found that ectopic expression of IL6 and ERβ were both significantly increased in malignant lung cancer, indicating a correlation between IL6 and ERβ in lung cancer. Higher IL6 or ERβ expression was associated with poor histopathological grade and metastasis in NSCLC patients. Importantly, both IL6 expression and ERβ expression were identified as independent prognostic factors for NSCLC, raising the possibility that IL6 and ERβ might be correlated with malignant behavior of lung cancer. As expected, our in vitro experiment demonstrated that E2 stimulation up-regulate IL6 and ERβ expression, enhancing the malignancy of NSCLC.

In this study, we focused on stimulation by E2, which is increasingly considered an important trigger of lung cancer progression [3]. We demonstrated that E2 induces ERβ activation and subsequent up-regulation of IL6 expression. The increase of IL6 expression included both intracellular IL6, detected by western blot, and secreted IL6, detected by ELISA.

There exist two mechanisms of action for E2 interaction with ERs, genetic and non-genetic [12, 38, 39, 46]. In our study, we emphasize the non-genetic pathway because the AF-1 domain in ERβ is less functional [7, 40]. We found that activated ERβ might function in the cytosol via MAPK/ERK and PI3K/AKT pathways. Using the MEK inhibitor U0126 and the PI3K inhibitor LY294002 to treat lung cancer cells, we found that the MAPK/ERK and PI3K/AKT pathways are involved in the regulation of IL6 expression. As for genomic actions, we explored the IL6 promoter, searching for potential EREs using the JASPAR database (jaspar.genereg.net), and four putative sites were predicted (Additional file 5). Given that ERs usually act as transfection factors in cancer cells, the existence of potential EREs indicated that ERβ might contribute to IL6 expression in this way, which requires further investigation.

One of the salient features of our study is that we used two mouse models. To exclude the influence of endogenous female hormones, recipient mice were ovariectomized before treatment. First, we made a urethane-induced adenocarcinoma model to explore the mechanism of E2 stimulation of IL6 expression. In addition, because our previous study confirmed that subtypes of ERβ have differential function in NSCLC, we wanted to elucidate whether each ERβ subtype behaves differently with respect to up-regulation of IL6 expression. Results showed that E2 administrate activated ERβ-induced IL6 expression, promoting the growth of tumors. ERβ1 and ERβ5 are essential for the proliferative effect of E2.

As mentioned before, Wang et al. reported that Stat3 is a downstream signaling molecule of IL6 and can up-regulate the expression of ERβ [21]. Given the fact that our results confirmed that ERβ promoted IL6 expression via the MAPK/ERK and PI3K/AKT signaling pathways in lung cancer cells with E2 administration, we next tested whether there exists a positive feedback loop between IL6 and ERβ in lung cancer. Interestingly, Che et al. reported that IL6 induced ERβ expression via Stat3; however, in A549 cells stimulated with DPN (an ERβ agonist), they did not observe further phosphorylation of Stat3. These results may indicate that Stat3 regulation of ERβ is not bi-directional. An additional study confirmed that IL6 up-regulate the aromatase level and E2 concentration in stromal cells but not in endometrial cancer cells [20]. Similar results were observed in cervical carcinoma and NSCLC [47, 48]. In our study, we blocked IL6 signal with Stattic (a selective inhibitor of Stat3 which is the major signal pathway of IL6) rather than the usage of neutralizing antibodies specific for IL6 or IL6R components to further detected the relationship of ERβ/IL6/Stat3. We found E2 up-regulate IL6 expression and promote lung cancer. However when manage E2 with Stattic the effect weaken. Meanwhile further phosphorylation of Stat3 was observed in mouse tumor tissue and human lymph node metastases. Taken together, these results indicate that there is a positive feedback loop between IL6 and ERβ in lung cancer (Additional file 6: Figure S5) by an autocrine mechanism, where treatment with E2 induces IL6 expression. Secreted IL6 can then bind with IL6R to activate p-Stat3 or up-regulate aromatase expression to promote E2 synthesis. These specific mechanisms remain to be further investigated. Our results highlight the possibility of a synergistic effect in treating lung cancers through a combination of ERβ and IL6 inhibitors.

## Conclusions

In conclusion, our results evidently reveal that E2 up-regulated IL6 expression through the ERβ pathway. Ectopic expression of IL6 stimulated by E2 contributes to a poor outcome in lung cancer. ERβ isoforms play differential roles in E2-stimulated IL6 expression, with ERβ1 and ERβ5 being essential for E2’s proliferative effect. Autocrine IL6 and ERβ may represent a positive feedback loop, and synergistic inhibition of ERβ and IL6 may serve as a potential target for prognostic assessment and therapeutic intervention in lung cancer.

## Additional files

Additional file 1: **Figure S1.** (A) IL6 and ERβ were not present in pneumocytes of normal lung tissues. (B) IL6 and ERβ high and low expression in tumor cases. For comparison between two groups, the χ2 test was applied. Significance of mean differences in staining scores between “Well/Moderate” and “Poor” tumor differentiation grade groups. (C) The significance of mean differences in staining scores between “Metastasis” and “No Metastasis” groups is shown. (D) Kaplan–Meier OS curves (http://www.kmplot.com/lung) of 1926 lung cancer patients. The overall survival (OS) rate in patients with high IL6 expression was significantly lower than that in patients with no or low IL6 expression (probe 205207_at). ERβ high expression indicates a shorter OS (probe 210780_at). Kaplan–Meier OS curves (http://www.kmplot.com/lung) of 866 lung adenocarcinomas and 675 squamous carcinomas show that IL6 expression is related to adenocarcinoma, but not to squamous carcinoma (probe 205207_at). (TIF 2213 kb)

Additional file 2: **Figure S2.** Upregulation of IL6 by E2 treatment regulates aggressiveness H1793 tumor cells. Autocrine IL6 was analyzed by ELISA assay after concentration-dependent treatment with E2 or Ful in H1793 cells. (B) Upregulation of IL6 by E2 or Ful was determined by immunofluorescence in H1793 cells. (C) Colony formation assay measuring the proliferative activity in H1793 cells. (D) Wound-healing assays were performed to assess NSCLC cell H1793 migration. Wound closure was determined 24 h after the scratch. (E) Transwell assay was used to quantify H1793 migration and invasion capacity. The average number of cells per field of view is plotted in three different experiments. (E) ELISA detection of the effect of E2 and its receptor antagonist Ful on IL6 expression and influence of the MEK inhibitor U0126 (60 nM) or a selective PI3K inhibitor of LY294002 (0.6 uM) on E2-mediated IL6 expression through MEK/ERK and PI3K/AKT activation in H1793 cells. (TIF 8517 kb)

Additional file 3: **Figure S3.** E2 regulates IL6 expression through ERβ and affects the malignancy of lung cancer cell H1793. (A) Autocrine IL6 was analyzed by ELISA assay after overexpression or knockdown of ERβ in H1793 cells. (B) Upregulation of IL6 by E2 was determined by immunofluorescence in H1793 cells. (C) Colony formation assay measuring the proliferative activity in H1793 cells after overexpression or knockdown of ERβ. (D) Wound-healing assays were performed to assess H1793 cell migration in response to modified ERβ expression. (E) Transwell assay was used to quantify cell migration and invasion capacity with respect to the ERβ expression level in H1793 cells. (TIF 7627 kb)

Additional file 4: **Figure S4.** (A) Immunofluorescence was used to detected expression of IL6 and ERβ in murine lung tumors. (B) A549 cells visualized with fluorescence microscopy detection of the GFP fluorescence of shRNA lentiviral particles. (C) Western blot verification of transfection efficiency. (TIF 9771 kb)

Additional file 5: IL6 promoter sequence and four putative EREs predicted by the JASPAR database (jaspar.genereg.net). (TIF 919 kb)

Additional file 6: **Figure S5.** Illustration of a positive feedback loop involving IL-6 and E2 promoting the growth of lung cancer by autocrine mechanisms. E2 stimulates IL6 expression through ERβ activation followed by downstream MAPK/ERK and PI3K/AKT pathway activation, which in turn confers ERβ expression. (DOCX 67 kb)

## Abbreviations

AF-1: activating function-1
AKT: PKB (protein kinase B)
CCK8: cell counting kit
E2: estrogen
ELISA: enzyme-linked immunosorbent assay
ERK: extracellular regulated protein kinases
ERβ: estrogen receptor beta
Ful: Fulvestrant
GFP: Green fluorescent protein
HRT: hormone replacement therapy
IL6: Interleukin 6
MAPK: mitogen activated protein kinase
NSCLC: Non-small cell lung cancer
OS: overall survival
PI3K: phosphoinositide 3-kinase
STAT3: signal transducer and activator of transcription 3
TMA: tissue microarray
