# Supplementary figures and images for: 17β-estradiol upregulates IL6 expression through the ERβ pathway to promote lung adenocarcinoma progression
Source: J Exp Clin Cancer Res. 2018 Jul 3;37:133. doi: 10.1186/s13046-018-0804-5 (PMC6029357; doi:10.1186/s13046-018-0804-5)

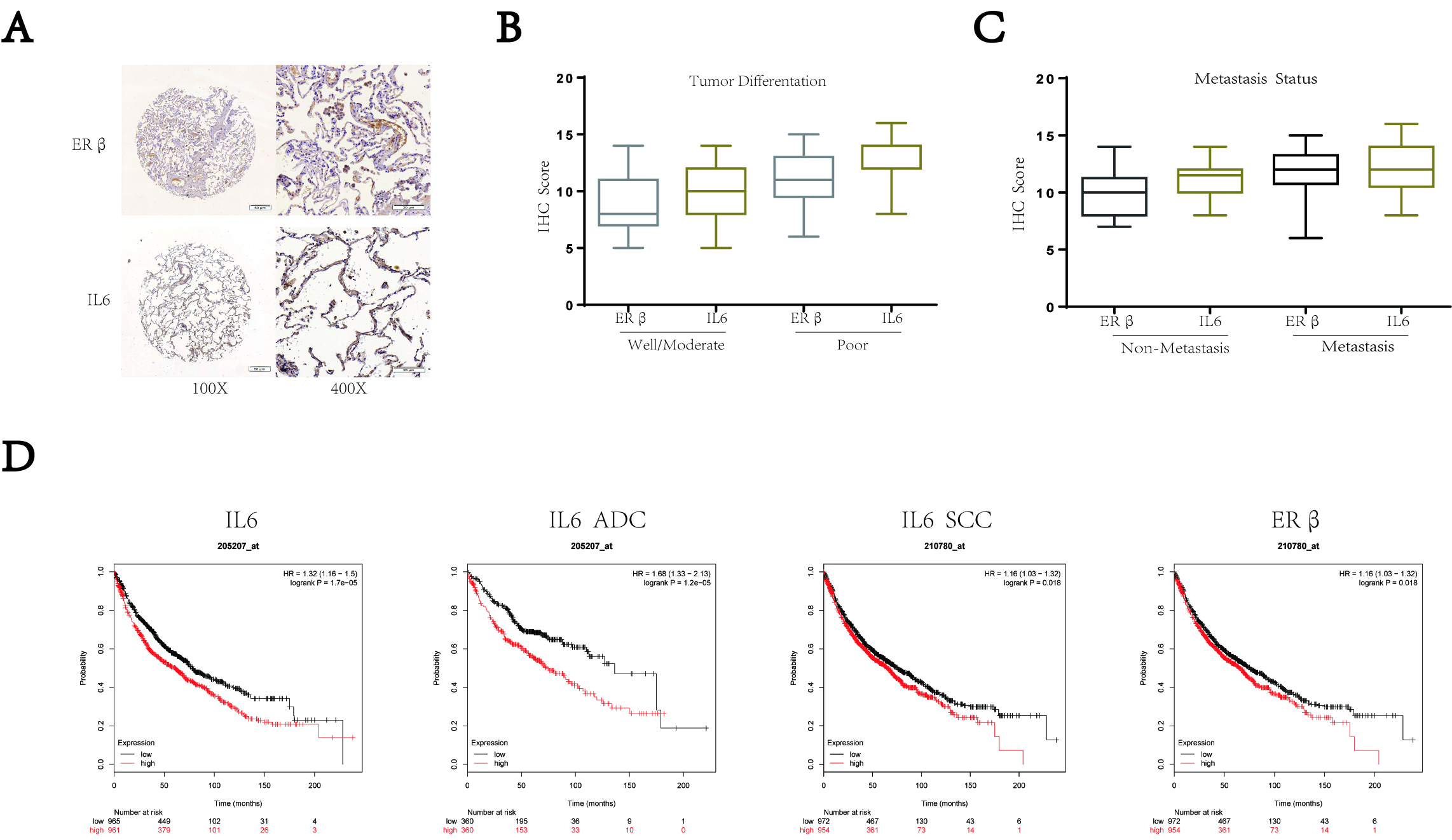

Supplement: Supplementary file 1 — Figure S1. (A) IL6 and ERβ were not present in pneumocytes of normal lung tissues. (B) IL6 and ERβ high and low expression in tumor cases. For comparison between two groups, the χ2 test was applied. Significance of mean differences in staining scores between “Well/Moderate” and “Poor” tumor differentiation grade groups. (C) The significance of mean differences in staining scores between “Metastasis” and “No Metastasis” groups is shown. (D) Kaplan–Meier OS curves (http://www.kmplot.com/lung) of 1926 lung cancer patients. The overall survival (OS) rate in patients with high IL6 expression was significantly lower than that in patients with no or low IL6 expression (probe 205207_at). ERβ high expression indicates a shorter OS (probe 210780_at). Kaplan–Meier OS curves (http://www.kmplot.com/lung) of 866 lung adenocarcinomas and 675 squamous carcinomas show that IL6 expression is related to adenocarcinoma, but not to squamous carcinoma (probe 205207_at). (TIF 2213 kb) [file 13046_2018_804_MOESM1_ESM.tif]

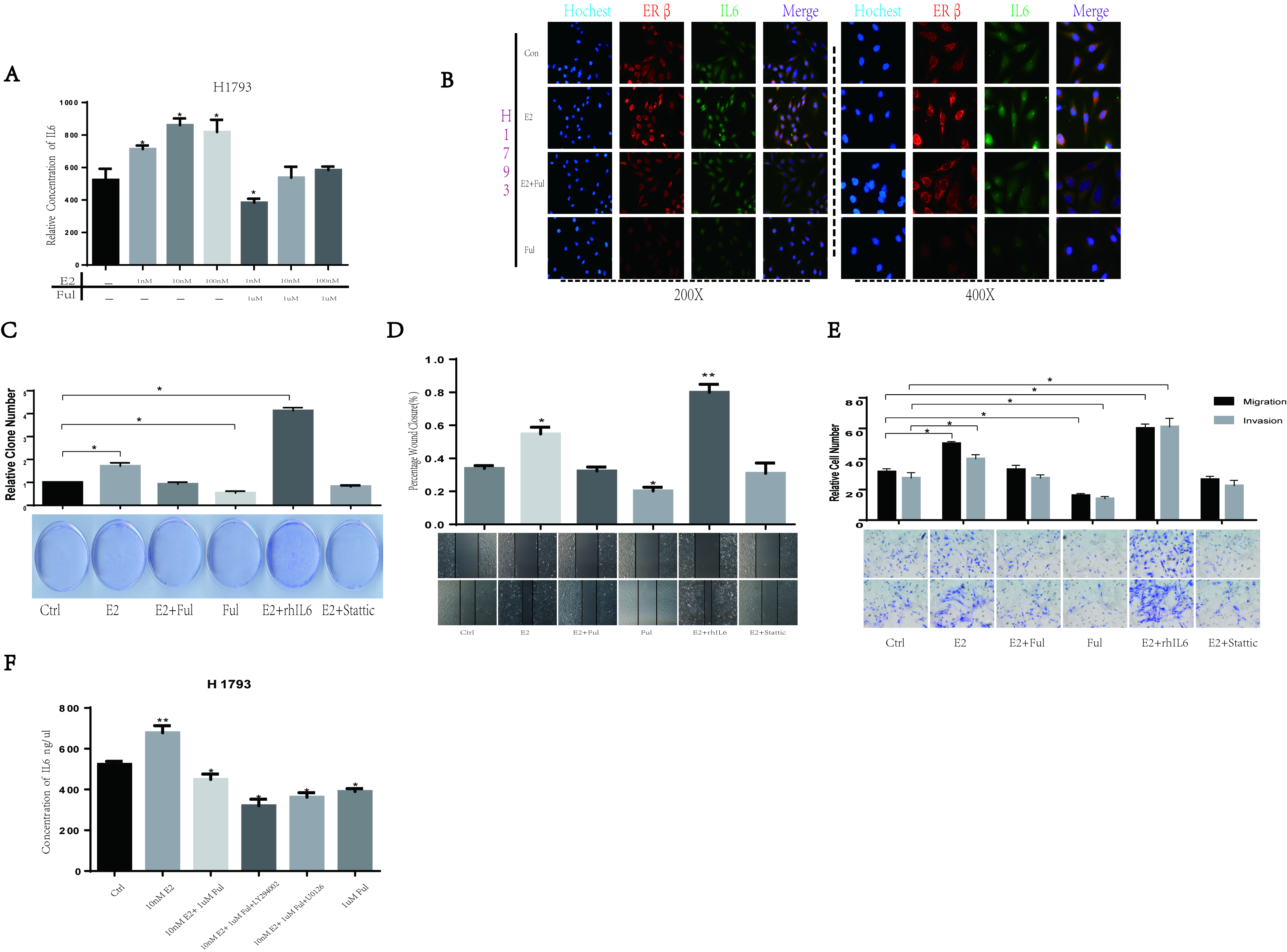

Supplement: Supplementary file 2 — Figure S2. Upregulation of IL6 by E2 treatment regulates aggressiveness H1793 tumor cells. Autocrine IL6 was analyzed by ELISA assay after concentration-dependent treatment with E2 or Ful in H1793 cells. (B) Upregulation of IL6 by E2 or Ful was determined by immunofluorescence in H1793 cells. (C) Colony formation assay measuring the proliferative activity in H1793 cells. (D) Wound-healing assays were performed to assess NSCLC cell H1793 migration. Wound closure was determined 24 h after the scratch. (E) Transwell assay was used to quantify H1793 migration and invasion capacity. The average number of cells per field of view is plotted in three different experiments. (E) ELISA detection of the effect of E2 and its receptor antagonist Ful on IL6 expression and influence of the MEK inhibitor U0126 (60 nM) or a selective PI3K inhibitor of LY294002 (0.6 uM) on E2-mediated IL6 expression through MEK/ERK and PI3K/AKT activation in H1793 cells. (TIF 8517 kb) [file 13046_2018_804_MOESM2_ESM.tif]

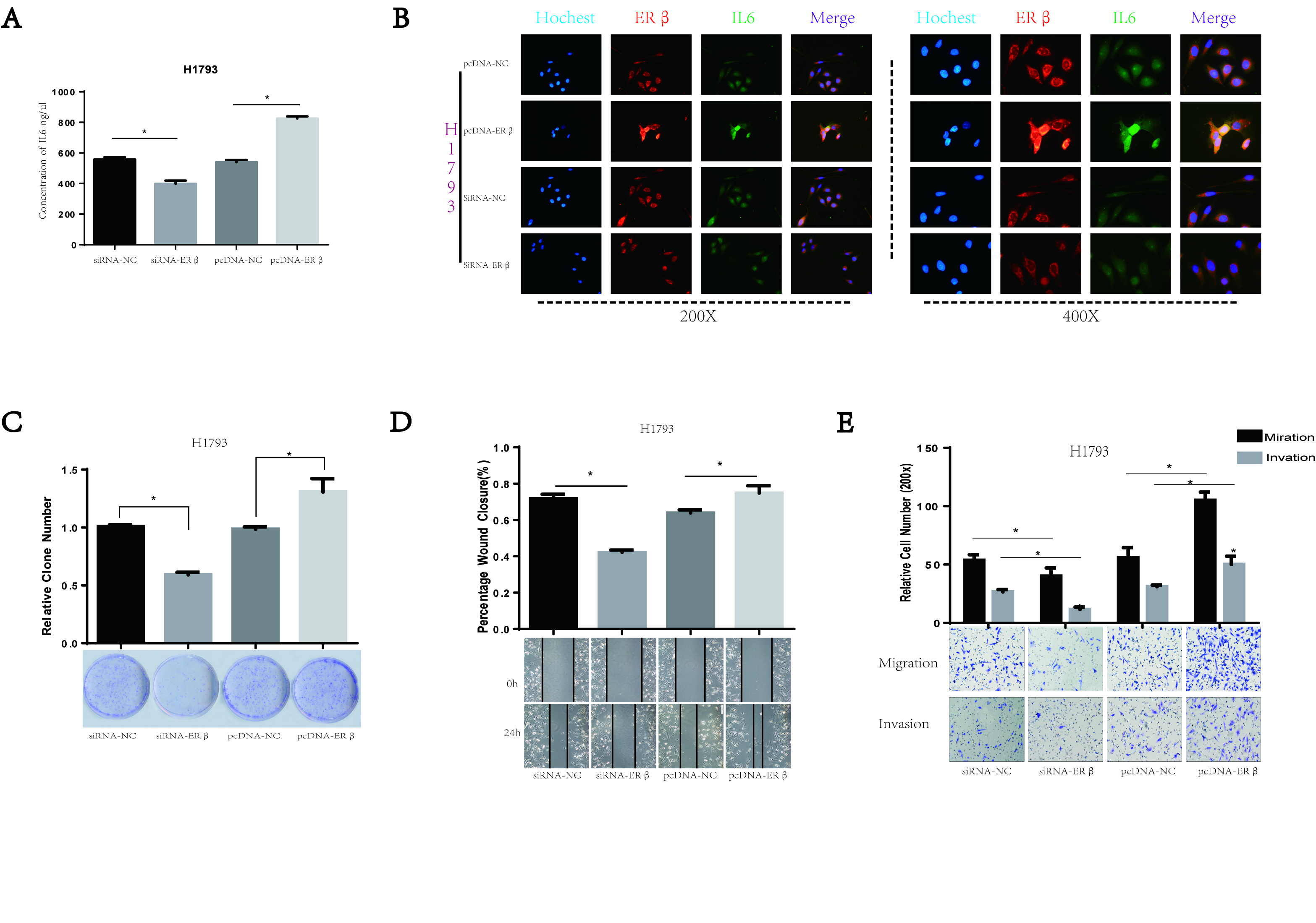

Supplement: Supplementary file 3 — Figure S3. E2 regulates IL6 expression through ERβ and affects the malignancy of lung cancer cell H1793. (A) Autocrine IL6 was analyzed by ELISA assay after overexpression or knockdown of ERβ in H1793 cells. (B) Upregulation of IL6 by E2 was determined by immunofluorescence in H1793 cells. (C) Colony formation assay measuring the proliferative activity in H1793 cells after overexpression or knockdown of ERβ. (D) Wound-healing assays were performed to assess H1793 cell migration in response to modified ERβ expression. (E) Transwell assay was used to quantify cell migration and invasion capacity with respect to the ERβ expression level in H1793 cells. (TIF 7627 kb) [file 13046_2018_804_MOESM3_ESM.tif]

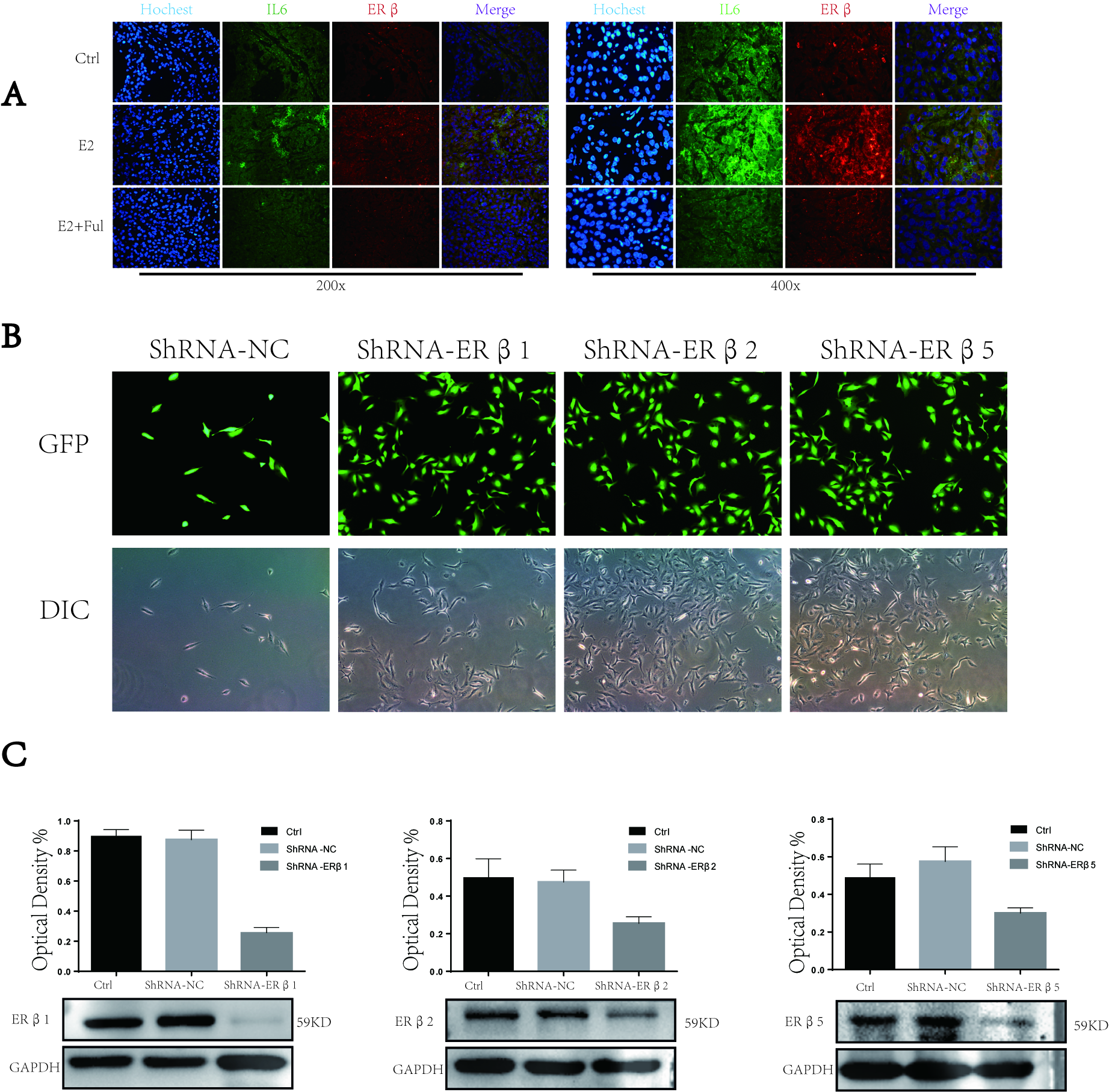

Supplement: Supplementary file 4 — Figure S4. (A) Immunofluorescence was used to detected expression of IL6 and ERβ in murine lung tumors. (B) A549 cells visualized with fluorescence microscopy detection of the GFP fluorescence of shRNA lentiviral particles. (C) Western blot verification of transfection efficiency. (TIF 9771 kb) [file 13046_2018_804_MOESM4_ESM.tif]

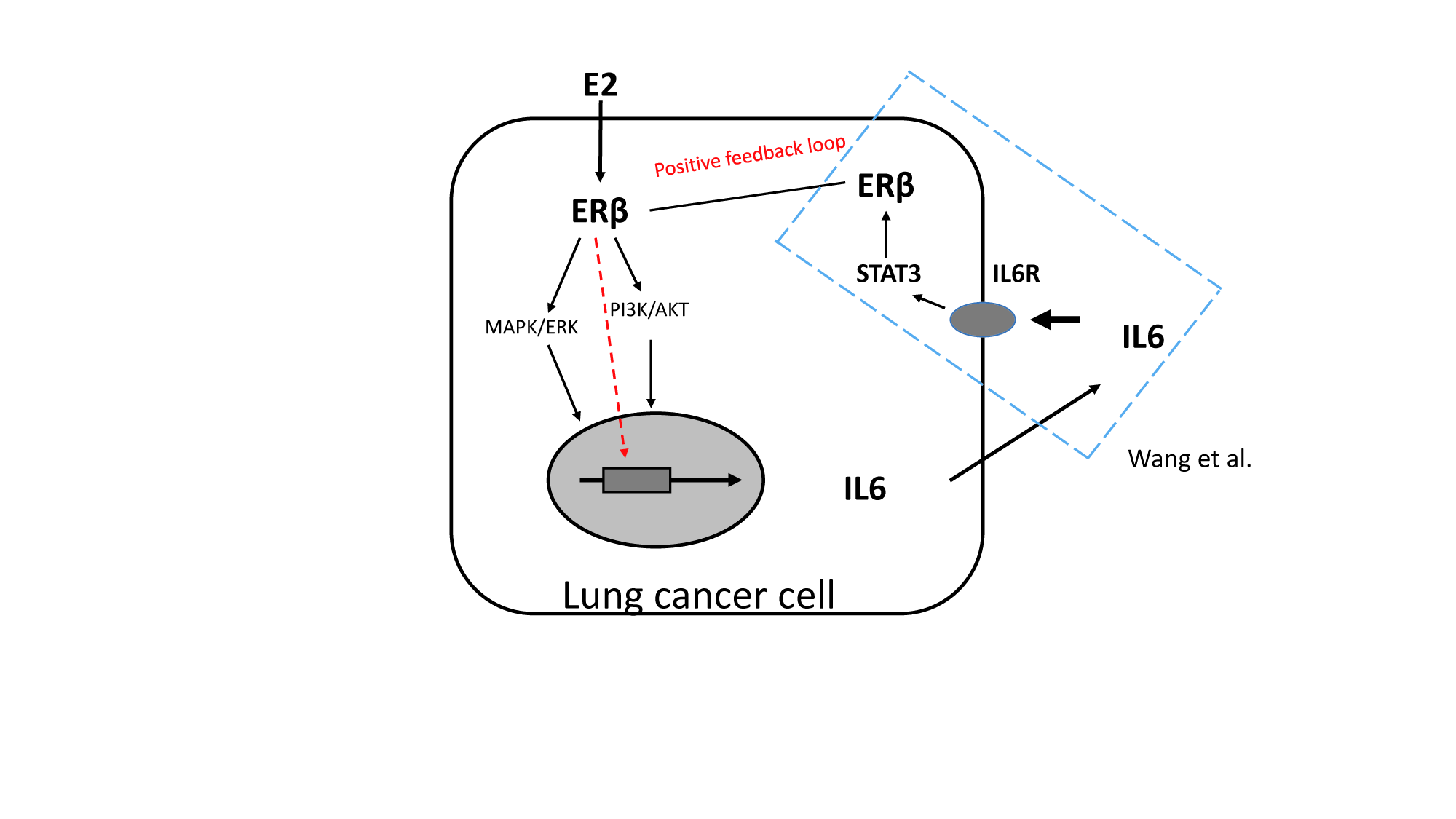

Supplement: Supplementary file 5 — IL6 promoter sequence and four putative EREs predicted by the JASPAR database (jaspar.genereg.net). (TIF 919 kb) [file 13046_2018_804_MOESM5_ESM.tif]
