# Supplementary material for: 17β-estradiol upregulates IL6 expression through the ERβ pathway to promote lung adenocarcinoma progression
Source: J Exp Clin Cancer Res. 2018 Jul 3;37:133. doi: 10.1186/s13046-018-0804-5 (PMC6029357; doi:10.1186/s13046-018-0804-5)

IL6 promoter sequence and four putative ERE predicted by the JASPAR database (jaspar.genereg.net)

>5' Flanking sequence chromosome:GRCh38:7:22723884:22725883:1

ACTTCACATCAGCTATGATGCAATCCAGCAACTAAAGTATTAGTTAATAAATGCTGACAG

CACAGCCTTTTCTGGTCACGTATTCATACTAAAATACGGGGGAGAGTTGGGGGGAGAGGG

GGATATATGGGAAATCTCTGTACCTTCCTCTCCATTTTGCTATGACCTAAAGCTGCCCTT

TAAAAAATACAAGGGGCTGGGCACAGTGGTTCACGCCTGTAAACCCAGCACTTTGGGAGG

CCGAGGCGCGTGGATCACCTGAGGTCAGGAGTTCAAGACCCGCCTGGCCAACATGGCAAA

ACCCCGTTTCTACTAAAAATACAAAAAGTAGCTGGGCGTGGTCGCATGCATCTGTAGTCC

CAGCTACTCAGGAGGCTGAGGCAAGAGAATTGCTTGAACCTGGGAGGCGGCGGTTGAAGT

GAGCCAAGATCATGCCATTGCCCTCCAGCCTGGGCAACAGAGCAAGACTCCTTCTCAAGA

GAAAAAACAAAACAAAACAAGAAAAAACAAAGAATGAGCTCTCCACGCGAAAAATCCATT

GAGATGCAAAGGAAGGAAGCTATCATTGTGGAATTGCACATGTCAGTTACATTAACGTTT

TTGGAGCAAGGTAGAGCTCATCTCTCCCACAAGCAAATTCCAGCCCAAAGCATTGATACT

AATAAAGTGCCATGCTGCGATGTGCAGGGGGCAGACAGTGTCTCCAAGCTCCCTACACAC

ATGCCTTCCCACAGTTTGCCCTTTCTTGACCCCAGAAGCATCAGGCCCCTTCACCCTCGA

GGGCCACTATCAGGAGTTTGAATTAATGGCAATCACCATGCACAGGGAAGGCTGTGGAAT

TCTGACATAAAAACACTTAGTGGAGGGCTTGGAAAAAGTCTAGTAGGAGCAAGACGCAAG

CTGGACTAATTATCTAAAACAAGAGACCTGGTTTGGGGATCTTAATGTTCTCAAAAAAGA

AAATTATTATTATTTTTCATTTTGCACTTTGTGCCATAAAACATTTTCAACAAAACATAG

AATCTCATTTCTTTTGAGGGAAAATGATTGGGAGACCAGCTCATTGCTGGCACAGAGGCC

TGGTTCATTCATAATTCCTTCATAGGCAAGACACCAGGTGAACCGATATAGCCGAGCTGG

AAGAGCTCTCCAAGGCAGAGACTCTGAGCCAAGGAATGTTCAAAGAGCTAGCATGTATTG

TGGGATTACTATGCGCCAGGAATTTTTTACACTGCATCACGTTCCATCTTCACAACAGCC

CTAGAAAGGAAGAACTATTATTACCCCCGTTTTATAGGTGAATAAACAAGGGCACAGGTC

CTTGATGTAACAGCCAGGATCAAACAGCTGGGAAGACGAGAAAACCTTTCCCAGGCTAGG

ATAACAGAGGATTTGGTTGAAAATACAGGCAATTAGGTGCTACCTCTGGGAAAAGGGGCC

AGGAGAGGAAGGAGACACTTTTCCCTGCATGCCCTGATGTCCTATTTGAACATTTTATCA

TGAACACGAACTTCCTATTTAAAAAACACTTTTTATTGAAAAGATAAATCTGTGTGTTGT

ATTGTGTCACTCAGTTCAAGTACTTGAAATTTATTGAATTGTATTTTCTAAAAAATAGAT

AGTTGAGTAAAAGCAAGCTCACATTACATAGACGGATCACAGTGCACGGCTGCGGAGCTG

GGAGCAGTGGCTTCGTTTCATGCAGGAAAGAGAACTTGGTTCAGGAGTGTCTACGTTGCT

TAAGACAGGAGAGCACTAAAAATGAAACCATCCAGCCATCCTCCCCCATTTTCATTTTCA

CACCAAAGAATCCCACCGCGGCAGAGGACCACCGTCTCTGTTTAGACAATCGGTGAAGAA

TGGATGACCTCACTTTCCCCAACAGGCGGGTCCTGAAATGTTATGCACGAAACAAAACTT

GAGTAAATGCCCAACAGAGGTCACTGTTTTATCGATCTTGAAGAGATCTCTTCTTAGCAA

AGCAAAGAAACCGATTGTGA


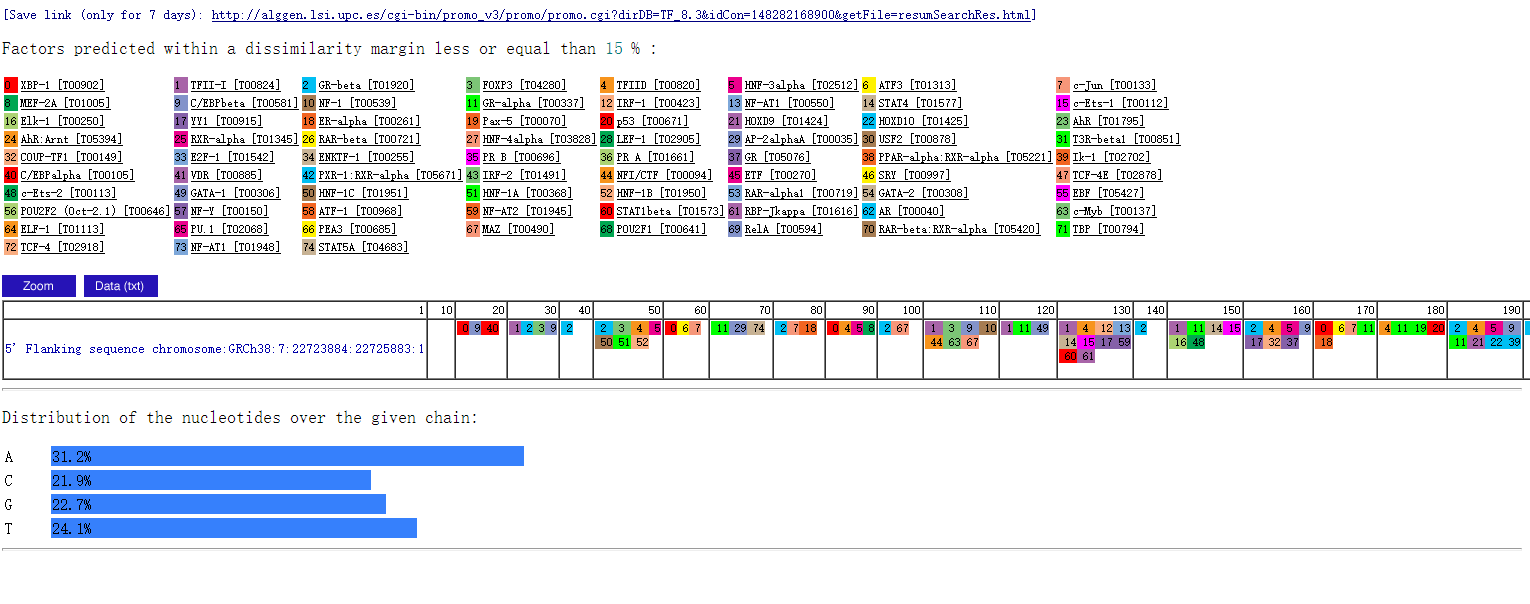

Supplement: Supplementary file 6 — Figure S5. Illustration of a positive feedback loop involving IL-6 and E2 promoting the growth of lung cancer by autocrine mechanisms. E2 stimulates IL6 expression through ERβ activation followed by downstream MAPK/ERK and PI3K/AKT pathway activation, which in turn confers ERβ expression. (DOCX 67 kb) [file 13046_2018_804_MOESM6_ESM.docx]
